# An Overview of Transcriptional Responses of Schistosome-Susceptible (M line) or -Resistant (BS-90) *Biomphalaria glabrata* Exposed or Not to *Schistosoma mansoni* Infection

**DOI:** 10.3389/fimmu.2021.805882

**Published:** 2022-01-12

**Authors:** Lijun Lu, Lijing Bu, Si-Ming Zhang, Sarah K. Buddenborg, Eric S. Loker

**Affiliations:** ^1^ Center for Evolutionary and Theoretical Immunology, Department of Biology, University of New Mexico, Albuquerque, NM, United States; ^2^ Wellcome Sanger Institute, Wellcome Genome Campus, Hinxton, United Kingdom

**Keywords:** transcriptomics, RNA-sequencing, *Biomphalaria glabrata*, *Schistosoma mansoni*, schistosomiasis, vector biology, comparative immunology

## Abstract

**Background:**

We seek to provide a comprehensive overview of transcriptomics responses of immune-related features of the gastropod *Biomphalaria glabrata* (Bg) following exposure to *Schistosoma mansoni* (Sm), a trematode causing human schistosomiasis. Responses of schistosome-susceptible (M line, or SUS) and -resistant (BS-90, or RES) Bg strains were characterized following exposure to Sm for 0.5, 2, 8 or 40 days post-exposure (dpe).

**Methods:**

RNA-Seq and differential expression analysis were undertaken on 56 snails from 14 groups. We considered 7 response categories: 1) constitutive resistance factors; 2) constitutive susceptibility factors; 3) generalized stress responses; 4) induced resistance factors; 5) resistance factors suppressed in SUS snails; 6) suppressed/manipulated factors in SUS snails; and 7) tolerance responses in SUS snails. We also undertook a gene co-expression network analysis. Results from prior studies identifying schistosome resistance/susceptibility factors were examined relative to our findings.

**Results:**

A total of 792 million paired-end reads representing 91.2% of the estimated 31,985 genes in the Bg genome were detected and results for the 7 categories compiled and highlighted. For both RES and SUS snails, a single most supported network of genes with highly correlated expression was found.

**Conclusions:**

1) Several constitutive differences in gene expression between SUS and RES snails were noted, the majority over-represented in RES; 2) There was little indication of a generalized stress response shared by SUS and RES snails at 0.5 or 2 dpe; 3) RES snails mounted a strong, multi-faceted response by 0.5 dpe that carried over to 2 dpe; 4) The most notable SUS responses were at 40 dpe, in snails shedding cercariae, when numerous features were either strongly down-regulated indicative of physiological distress or parasite manipulation, or up-regulated, suggestive of tolerance or survival-promoting effects; 5) Of 55 genes previously identified in genome wide mapping studies, 29 (52.7%) were responsive to Sm, as were many familiar resistance-associated genes (41.0%) identified by other means; 6) Both network analysis and remarkably specific patterns of expression of lectins and G protein-coupled receptors in categories 4, 6 and 7 were indicative of orchestrated responses of different suites of genes in SUS or RES snails following exposure to Sm.

## Introduction

Invertebrates play essential roles in supporting the life cycles of a wide range of viral, bacterial, and eukaryotic pathogens of humans and wild and domestic vertebrate hosts. Our knowledge of the roles played by invertebrate hosts, including a variety of arthropods and molluscs in both facilitating and resisting pathogen development, remains in need of further study with modern methods (1). This is particularly true for the freshwater gastropods that serve as obligatory hosts for the larval stages of digenetic trematodes or flukes, especially those involved in causing widespread neglected tropical diseases like schistosomiasis and fascioliasis. Insofar as control of such snail-borne diseases has traditionally emphasized chemotherapy to kill the adult worms living in the vertebrate definitive hosts, and often control falls considerably short of the mark of actually interrupting transmission, new methods of control are needed (2–4). The snail hosts or the parasite larval stages developing within snails are often considered as possible new targets to augment our control efforts.

A general impediment to pursuing new control efforts targeting snails or parasite life cycle stages occurring within snails has been the lack of a broader and deeper understanding of their transcriptomic repertoires, including how they react to one another, e.g. the interactome (5, 6). An important model system in this regard is the freshwater Neotropical pulmonate *Biomphalaria glabrata* (Bg) and the widely distributed agent of intestinal schistosomiasis, *Schistosoma mansoni* (Sm) (7–10). Bg is the most important intermediate host for Sm in the Neotropics (11). Genome sequences are available for both organisms (12, 13).

As is typical of the phylum Mollusca, the gastropod Bg has an innate immune system featuring circulating phagocytic cells called hemocytes, usually of two general types (granulocytes and hyalinocytes) that, in addition to phagocytosing bacteria, can also cooperate to encapsulate and kill larger pathogens like parasite larvae using oxidative and other killing mechanisms (9, 14–16). Circulating or hemocyte-associated lectins, which can be surprisingly diverse in variety, can opsonize immune targets and enhance the efficacy of hemocytes. Ability to discriminate among specific kinds of pathogens is evident. Some complement-like components are present as are Toll-like receptors, NF-κB transcription factors and distinctive repertoires of antimicrobial proteins. Heightened responses can be induced upon repeated exposure and some innate memory responses have been attributed to molluscs (12).

One important advantage for Bg is the availability of strains that are either susceptible or resistant to commonly maintained lab strains of Sm, which provide a fruitful means of identifying key host genes involved in resistance to infection or that are essential for promoting schistosome development in snails. Among the susceptible strains commonly employed are the NMRI and M line strains, the derivations of which were reviewed by Lewis et al. (17) and Sullivan (18). The M line Bg strain we used as a model susceptible host strain was originally derived by Newton (19) from a cross between albino Brazilian snails refractory to Sm, and pigmented wild-type schistosome-susceptible snails from Puerto Rico (20). The M line strain is susceptible to infection with the PR-1 isolate of Sm used in this study (17). PR-1 Sm was originally collected from infected snails in Puerto Rico in 1950 and has been maintained under NIH contract at the Biomedical Research Institute (Rockville, Maryland), and in our laboratory since 1985. With respect to resistant strains, two have been commonly studied in recent years including 13-16-R1 (20–23) and BS-90 snails (24–35). The BS-90 snails were originally derived from the field in Salvador, Bahia, Brazil by Paraense and Correa (36) and obtained from Dr. Paraense and brought to UNM in the late 1980’s. BS-90 snails can be infected by the LE strain of *S. mansoni* (37) but are resistant to others including PR-1 *S. mansoni* used here, miracidia of which are encapsulated within hours of penetration and killed (38). In addition, others have selected lines of Bg resistant to infection with echinostome trematodes (39–44).

Many comparisons of susceptible and resistant strains of Bg have been undertaken, involving examination of both constitutive or inherent differences (22, 30, 45–47), and how the strains differ in their responses to experimental exposure to a variety of insults including bacteria, molluscicides and trematodes (45, 46, 48, 49), often but not always Sm. Because this ever-expanding list of studies has turned up a long list of factors potentially associated with resistance or susceptibility to infection, we have summarized their results and candidates (see **Table S1**), both to facilitate our own subsequent analyses and also to aid others in gaining a full appreciation of literature involved. Methods of investigation have been diverse, ranging from general responsiveness to infection as measured by the proportion that develop cercariae-producing (patent) infections or by the extent of cellular responses as observed from *in vivo* or *in vitro* histological studies (50, 51). Circulating snail hemocytes have been extensively compared with respect to numbers or spreading ability (22), surface markers, enzyme content and expression levels of known immune factors (28, 48). Likewise, the humoral components of the immune responses of resistant and susceptible snails have received considerable attention, both at the protein and at gene sequence and transcript levels (52–54). More recently genome-wide mapping studies have been undertaken to identify the genes in particular chromosome regions associated with resistance (55–58). In addition, functionally-oriented reverse genetics approaches using RNAi to identify genes contributing to resistance have become more commonplace (25, 28, 32, 59–62), and the recent documentation of successful use of CRISPR to alter the sequence of a gene involved in determining chirality in lymnaeid snails (63) holds considerable promise for further functional studies.

Our emphasis here is on high throughput sequencing techniques that have included a broad array of techniques including expressed sequence tags (27, 43, 44), ORESTES studies (35, 64), microarrays (45, 46, 48, 65–67) and RNA-Seq transcriptomics studies, the latter being the focus of the current investigation. The first Illumina-based study involving Bg identified 1,685 genes exhibiting differential expression after immune challenge with bacteria or yeast (49). RNA-Seq has also been used to identify Bg genes associated with a state of heightened innate immunity (62) or FREP (fibrinogen-related protein)-encoding genes from snails differing in their susceptibility to Sm (68). RNA-Seq studies have also now been undertaken with field-derived specimens of the major African vector snail *Biomphalaria pfeifferi* (6) and with the intermediate hosts for *Schistosoma japonicum*, *Oncomelania hupensis* (69, 70). Recently, RNA-Seq has been used to explore responses of lab-reared *Biomphalaria* to Sm or *Schistosoma rodhaini* from sympatric or allopatric sources (71), and was used in a study of early (0.5-16 hours) exposure to Sm in BS-90 Bg to explore the interactions of *BgPiwi* and the retrotransposon *nimbus* in influencing resistance (72). Most recently, single cell transcriptomics methods were applied to isolated granulocytes and hyalinocytes from M line and BS-90 snails not exposed to parasites (47).

However, yet to be presented is a systematic and comparative overview of the transcriptional profiles provided by RNA-Seq for both Sm-susceptible and -resistant strains of Bg, both before and following exposure to Sm infection at various time points (10). Because of the quantity of transcriptomics data generated, we first examined the presence in, and responses of Bg, with respect to their expanded AIG family of GTPases (frequently represented as GIMAPs) and noted differences between strains in their expression before and after exposure to Sm (73). We also have found marked strain differences with respect to expression of the 39 members of the FREP (fibrinogen-related protein) family in Bg (74).

**Figure 1:**
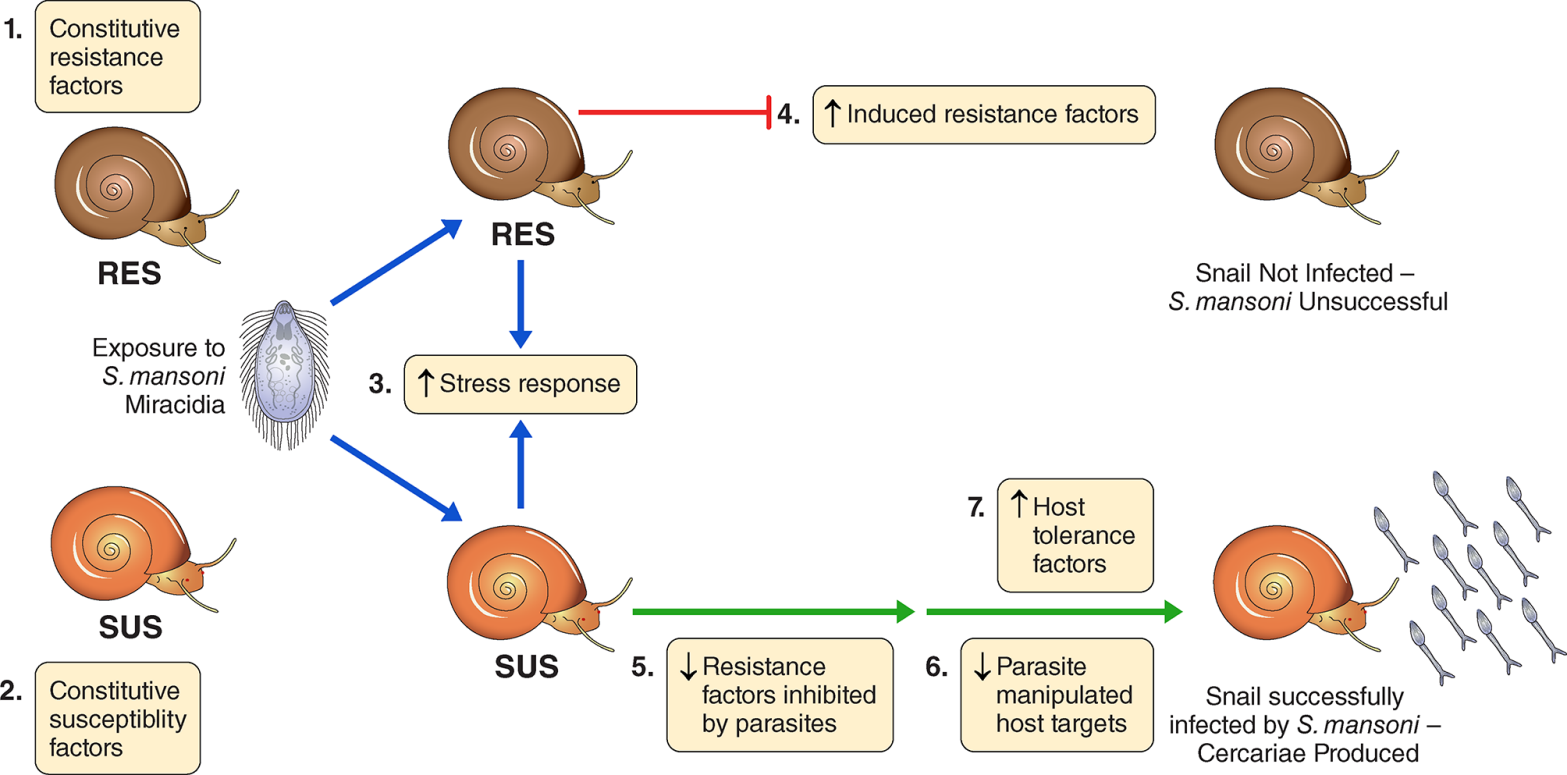
A conceptual overview for categorizing the transcriptomics responses of *B. glabrata* strains susceptible (SUS) or resistant (RES) to *S. mansoni*, exposed or not to *S. mansoni* infection. We identified 7 categories: 1) Constitutive resistance factors in RES (higher expression in unexposed RES than SUS); 2) Constitutive susceptibility factors in SUS (higher expression in unexposed SUS than RES); 3) Generalized stress responses in SUS and RES (genes up-regulated in both strains shortly following exposure, 0.5 and 2 dpe); 4) Induced resistance factors in RES (up-regulated in RES only at 0.5 or 2 dpe); 5) Resistance factors inhibited by parasites in SUS (down-regulated in SUS only at 0.5 or 2 dpe); 6). Possible parasite-manipulated host targets in SUS (down-regulated in SUS only at 8 or 40 dpe); 7) Host tolerance factors in SUS (up-regulated in SUS only at 8 or 40 dpe). We emphasize membership in any of the 7 categories should be validated by individual gene functional studies and we make no claim that these 7 categories provide exhaustive coverage of all possible responses or are necessarily mutually exclusive of one another.

Here we explore the effects of exposure to Sm on the *overall* transcriptomics responses of Bg, including consideration of the various candidates indicated in **Table S1**. **Figure 1** provides a conceptual approach for how we organized our thinking into seven different categories. It is offered with the understanding that the actual functional relevance of particular genes would of course require further functional validation. For example, we have attempted to spot constitutive differences among host strains in defense-related factors, components of putative stress responses that might be up-regulated in both snail strains following exposure to infection, or factors that might be conspicuously up-regulated uniquely in one strain or the other following exposure to infection. Responses of chronically infected snails may be indicative of parasite manipulation or host compensatory or tolerance responses. Taking into account the results of others, we provide lists of snail genes that seem to comprise the host side of the interactome, with those host-responsive aspects of the parasite genome that comprise the other part of the interactome to be provided in a separate study to follow.

## Materials and Methods

### Snails and *S. mansoni* Used

The Sm susceptible M line strain (19), hereafter referred to as “SUS,” and the resistant BS-90 (36) strain, hereafter referred to as “RES” of Bg were used through this paper. Snails of both strains were reared and maintained by using typical methods for lab colonies (75). The PR-1 strain of Sm (17) was used for all parasite exposures.

### Experimental Infections, RNA-Sequencing and Differential Expression (DE) Analysis

To investigate the overall expression levels of Bg SUS and RES snails with or without Sm exposure, a comprehensive transcriptomics study was carried out. Snails and experimental treatments used in this study are described in Lu et al. (73, 74). Briefly, RES or SUS juvenile snails (5-8mm diameter) were individually put in the wells of 24-well plates, in 2 ml artificial spring water and exposed to 20 PR-1 Sm miracidia per snail, for 6 hours. Control snails were treated similarly but were not exposed to miracidia. Snails of each group were moved to aerated aquaria containing artificial spring water (ASW) at 25-27°C and fed with lettuce. For both strains, snails exposed to Sm or time-matched control snails were sampled at 0.5, 2, 8 or 40 days post-exposure (dpe). The unexposed snails used as controls for the 2- and 8 dpe exposed snails were the same. The times selected corresponded to key stages of Sm development in the snail: early penetration when the transition from miracidium to mother sporocyst may still be underway (0.5 dpe); mother sporocyst establishment in snail head-foot and beginning of germinal cell proliferation (2 dpe); daughter sporocyst production in mother sporocysts (8 dpe); and full-fledged infection with daughter sporocysts in the snail digestive gland and production (shedding) of cercariae (40 dpe) (76). Snails sampled at 0.5, 2, and 8 dpe were juveniles whereas snails collected at 40 dpe had grown to be adults. For each strain and sampling time, 7-9 snails were collected. Snails sampled from this study were individually preserved in TRIzol reagent (Invitrogen) and stored at -80°C until extraction. The RNA extraction process followed TRIzol manufacturer’s instructions (Invitrogen) with modification of a few steps to yield more RNA (67). RNA samples were further purified and the quality and quantity of RNA extracted from each sample measured. To ensure the experimental snails used for sequencing were truly infected with Sm, a PCR assay was applied to verify Sm infection in each snail DNA sample. Sm specific *ND5* primers (77) were used to confirm the parasite infection in individual snails. Quality and quantity of RNA extracted from each sample were measured with a Nanodrop 2000c spectrophotometer (Thermo Fisher Scientific) and Agilent 2100 Bioanalyzer (Agilent RNA 6000 Pico kit), respectively. Replicates (3~6 snails per group per sampling time point) were selected for library preparation (overview of snail groups and biological replicates in **Table S2**; total of 56 snails sampled). Complementary DNA libraries were paired-end sequenced (2x150 base reads) on an Illumina NextSeq 500 instrument (Illumina). Based on the sequencing quality, raw reads were trimmed and filtered using Trimmomatic v0.36 (78) with slide window of 4 nt, average score above 20 and minimum length of 36 nt. Workflow of read trimming and mapping was build using Unix shell commands with application GNU-Parallel (79) to perform jobs in parallel. A posterior probability of differential expression (PPDE) ≥ 0.95 for EBSeq, or *P*-value ≤ 0.05 DESeq2 and EdgeR were set as cutoff for DE analysis. More detailed description of the library preparation, sequencing and DE analysis can be found in Lu et al. (73, 74). Three methods to analyze DE for control snails were used (DESeq2, EdgeR and EBSeq) and the overall patterns of DE revealed by each were similar (See **Figure S1**). We chose EBSeq to represent our results because of its greater sensitivity in detecting differential expression (DE) among less abundant transcripts.

### Annotation Updates for Uncharacterized Proteins

Several interesting genes were annotated as “uncharacterized protein” or “NA” (no annotation) in the Bg BB02 database (﻿v1.6). To obtain a more complete annotated DE gene list, all transcripts after mapping to the BB02 genome were reannotated using BLAST against multiple databases, including the NCBI non-redundant nucleotide database, non-redundant protein database, SWISS-PROT protein knowledgebase, and our own database of interesting mollusc- or gastropod-related genes manually collected from published literature in **Table S1**. Throughout, we consider genes in **Table S1** to be “previously-identified putative resistance factors”. BLAST hits were first filtered with minimum sequence identity of 70% (for BLASTn, and 30% for BLASTp) and E-value < 10^-5^ as cut-offs and then manually evaluated considering the percentage of aligned region to the query and subject sequence length. All uncharacterized genes were screened based on query coverage (qcov), subject coverage (scov) and query identity. Only those with qcov >70%, scov >70% and identity >30% were updated in our annotation and used for further analysis. To better understand the DE genes, InterPro (80), a database of protein sequence analysis and classification was used for predicting domains of genes. Transcript types (mRNA, non-coding RNA, pseudogenic_transcript, etc.) based on Vectorbase database (﻿v1.6) were added to each DE gene as well.

Additionally, we have examined the genes identified in four genome wide mapping studies found to be involved with resistance/susceptibility of Bg to Sm, relative to our transcriptomics results (**Table S3**). We also looked at six feature-specific studies for the sake of comparison (**Table S3**).

### Weighted Gene Co-Expression Network Analysis (WGCNA)

Biological systems tend to have modular structure and functionally related genes are commonly found within the same modules (81). Modules can be ﻿identified *via* hierarchical clustering of a weighted coefficient matrix. To investigate potential connections among genes and to narrow down target genes of interest, a signed (including positive and negative correlations) co-expression gene network was constructed using the R package WGCNA (82), with a total of 56 snail (28 RES and 28 SUS) gene expression datasets from this study. The normalized gene read counts from RES or SUS snails (and corresponding unexposed controls) were used in separate WGCNA analyses, taking into account expression and possible sequence differences between the two strains. The soft thresholding power setting used were “5” for the RES analysis and “20” for the SUS analysis, according to the network topology analysis function *pickSoftThreshold* in the WGCNA package. Network construction, and module detection using hierarchical clustering with a dynamic tree cut method were performed by using function *blockwiseModules* (with parameters TOMType = “signed”, minModuleSize = 30, mergeCutHeight = 0.25) (82).

For RES snails, to identify key modules that are significantly putatively associated with the phenotype resistant to Sm, the module eigengene (ME) was used to summarize the expression profiles of each module. The correlation of weighted Pearson correlation between “module” and “infection” was calculated with the infection quantified values assigned to all unexposed (control) RES as “0”, the Sm exposed RES snails assigned as “10” for 0.5 dpe, “9” for 2 dpe, “1” for 8 dpe, and “0” for 40 dpe, respectively.

Similarly, for SUS snails, to identify modules related to Sm infections, module-traits correlation tests were performed. All unexposed (control) SUS snail were assigned as “0”, and the Sm exposed SUS snails were assigned as “1” for both 0.5 and 2 dpe; “10” for 8 dpe, and “100” for 40 dpe.

For both strains, the module with the most significant correlation (Pearson correlation coefficient R^2^ and *P* value) for each strain was selected for further analysis. Co-expression gene networks in key modules from SUS or RES strains were visualized in Cytoscape 3.8.0 (83).

### Other Analyses

Venn diagrams were generated using R package VennDiagram v1.6.20. Principal Component Analysis (PCA) plots were generated using functions embedded in R package DESeq v1.22.1. Summarized tables and figures were generated using R base packages (84) and ggplot2 v3.1.0 (85).

## Results

We abbreviated the Vectorbase gene IDs for the sake of readability: for instance, “BGLB000152” is referred to as “Bg152”, “BGLB042374” is referred to as “Bg42374”, and so on.

A total of 56 snail samples (unexposed snail controls and snails exposed to Sm) were sequenced and used in this study. We mapped all sequencing reads to the Bg BB02 (12) and Sm (86) genomes separately, then counted the reads mapped uniquely to each genome and the reads shared by both genomes. The shared reads in each snail replicate were regularly at or under 1% of the total sequencing reads in the transcriptional dataset. Due to the low percentage of shared reads observed and the unknown impact of removing them, we kept the shared reads for mapping to the Bg genome for the DE analysis. A total of 792 million paired-end reads representing about 91.2% of the estimated 31,985 genes in the Bg genome were detected.

During the course of this investigation, in addition to information provided in the *B. glabrata* BB02 reference genome (﻿v1.6) (12), we undertook additional up-to-date sequence blast analyses for gene functional annotations. Based on the updated blast information, three main groups of transcripts were sorted: a) annotated transcripts; b) uncharacterized proteins (previously discovered, coding uncharacterized proteins); and c) “NA” (no annotation), including no blast hit to NCBI Non-Redundant (NR) database). For any “NA” gene, a particular transcript type was assigned, following the convention of the *B. glabrata* BB02 reference genome. These included mRNA, non-coding RNAs (ncRNA), pseudogene transcript, or others. A gene classified as “NA” is possibly first reported in the Bg BB02 genome considering it had no blast hits in the current NCBI NR database.

We also noticed cases where a particular annotated gene appeared to be both up- and down-regulated at the same time. For example, at 40 dpe in SUS snails vs. Sm, temptin transcripts Bg9018, Bg9019, Bg9020 and Bg26839 were all up-regulated, but temptin transcript Bg39942 was down-regulated. The former 4 temptin transcripts were 642~779bp in length, while temptin Bg39942 was only 456bp long. Similar patterns were also found for perlucin, alpha-amylase, hemocytin and others. Whether this is due to transcript variations from the same gene or reflects contributions of closely related members of a gene family deserves further study. It should be noted too that in some cases, as for example with hemocytin (Bg20186), a gene down-regulated at one time point (for instance, 8 dpe) would place it in our category 6 (**Figure 1**), might be upregulated at another time (say 40 dpe), resulting in its inclusion in category 7 as well.

### A Comparison of Constitutive SUS and RES Transcriptomes

#### Overview of Control Groups and RNA Raw Reads Obtained

**Figure 2:**
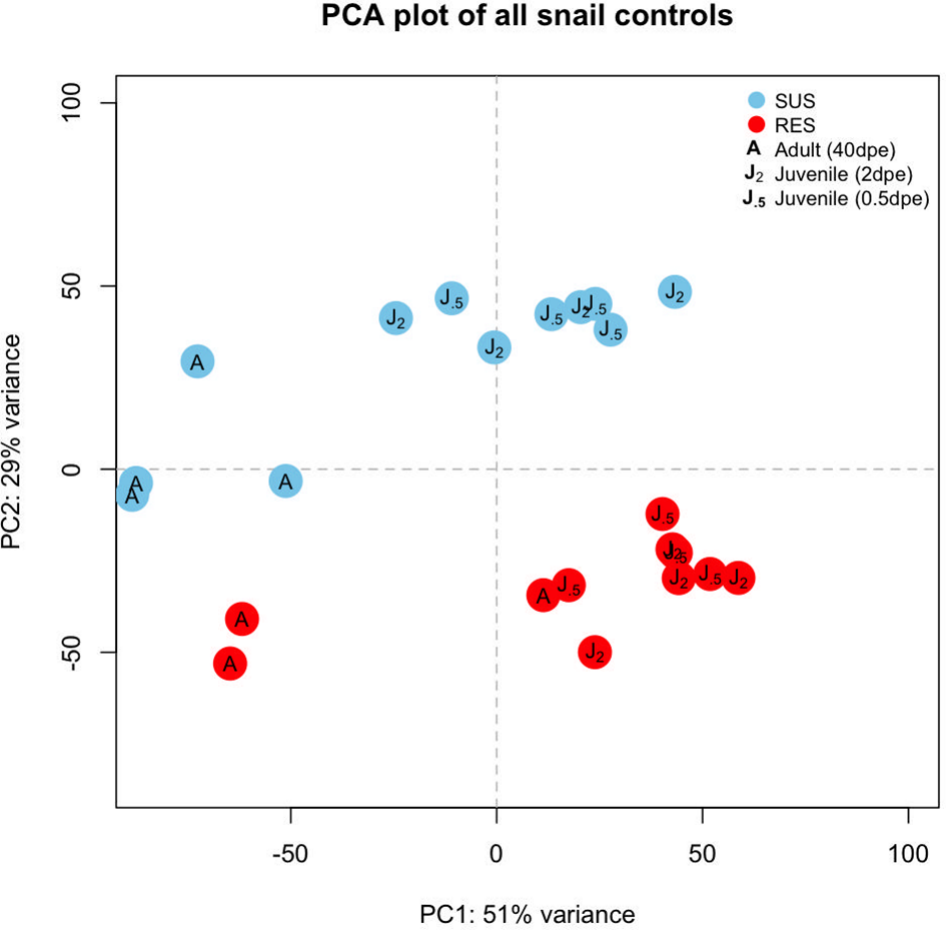
Principal components analysis (PCA) plot indicating the six groups of unexposed control snails examined in this study. Results for SUS snails are shown in blue circles and red circles indicate RES snails. “**J_.5_**” refers to juvenile control snails matched in size and time matched to snails exposed to *S. mansoni* for 0.5 days, “**J_2_**” refers to control juveniles matched to snails exposed to *S. mansoni* for 2 or 8 days, and “**A**” refers to adult snail size (time matched controls for snails exposed to Sm, 40 dpe).

Six groups of control snails not exposed to Sm were considered: three from SUS and 3 from RES, with each group containing 3-4 biological replicates (**Table S2**). Those snails indicated in the 40 dpe group were adults, the remainder were juveniles. Raw reads (~12 million unique and shared snail reads/snail sample) with a quality score above 20 and length of at least 36 nt (post-trimming) were mapped to the BB02 reference Bg genome (12) for differential expression analysis. A PCA plot provided an overview of expression profiles among the different control groups (**Figure 2**). In general, adults and juveniles tended to separate along PC axis 1, and members of the two strains separated along PC axis 2. The plot is indicative that RES and SUS snails have different overall constitutive patterns of gene expression.

#### An Overview of Differential Expression (DE) Among Unexposed Control RES and SUS Snails

**Figure 3:**
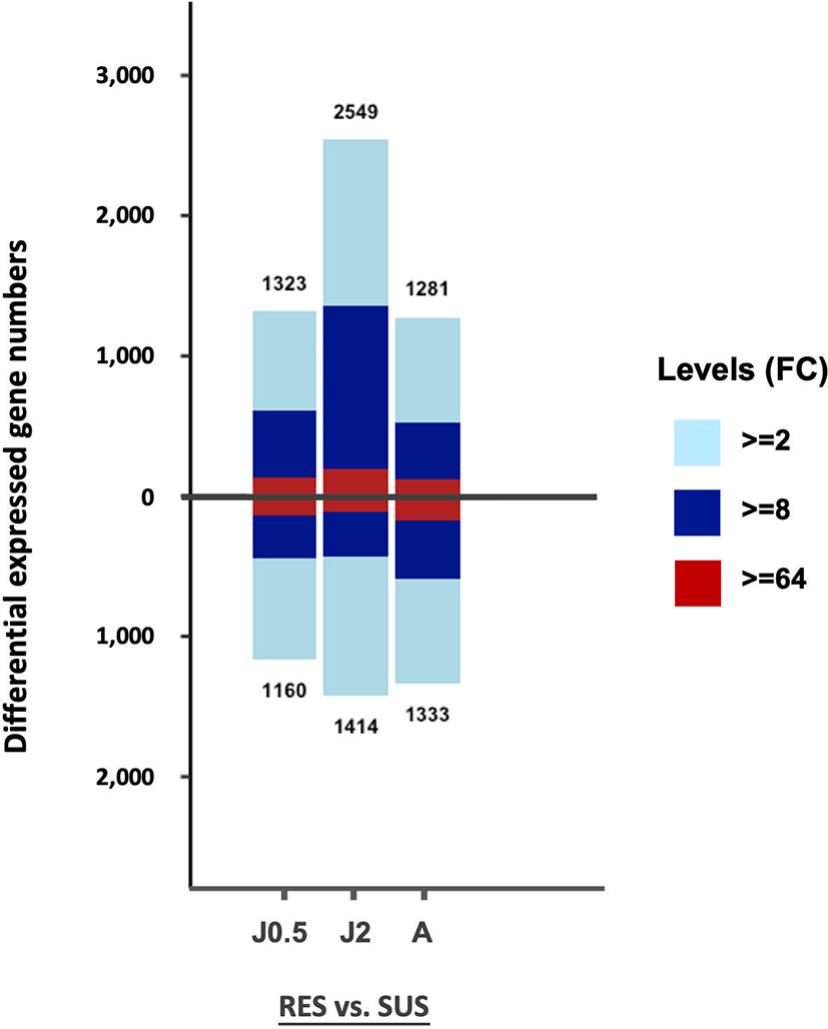
Bar graph showing all DE genes in the six different groups of control snails in **Table S2**. On the vertical axis, the number above the bar is the number of genes exhibiting an excess in transcripts in RES compared to SUS, and the number below the bar the number of genes with an excess in transcripts for SUS relative to RES. The three comparisons include RES vs. SUS with juvenile controls @0.5 dpe (J0.5), @2 dpe (J2), and with adult controls @40 dpe (A). The three colors in each bar indicate fold changes (FC) among comparisons: light blue 2-8; dark blue 8-64; and red greater than 64-fold.

The total number of DE genes identified for each of the 3 comparisons in **Table S2** is shown in **Figure 3**. Values above the base line indicate numbers of genes exhibiting an excess in transcripts for RES over SUS, and values below the line, the opposite. Only those genes with fold change (FC) values equal to or greater than 2 (FC ≥ 2), were taken into consideration for this and the following analyses.

The overall gene counts revealed significant differences in constitutive levels of gene expression between strains with the posterior probability of differential expression (PPDE) ≥ 0.95 in the EBSeq DE analysis. When enumerating *all* differentially expressed genes, for both comparisons involving juvenile snails, RES snails exceeded SUS snails, but for adult snails, SUS snails slightly outnumbered RES snails. For two of the three comparisons, the bars above and below the zero line were relatively similar, as might be expected of unexposed snails of two strains of the same species. Constitutive strain differences in expression of particular genes are investigated further below.

### Feature Specific Comparisons of Transcripts in Unexposed Control SUS and RES Snails

**Figure 4:**
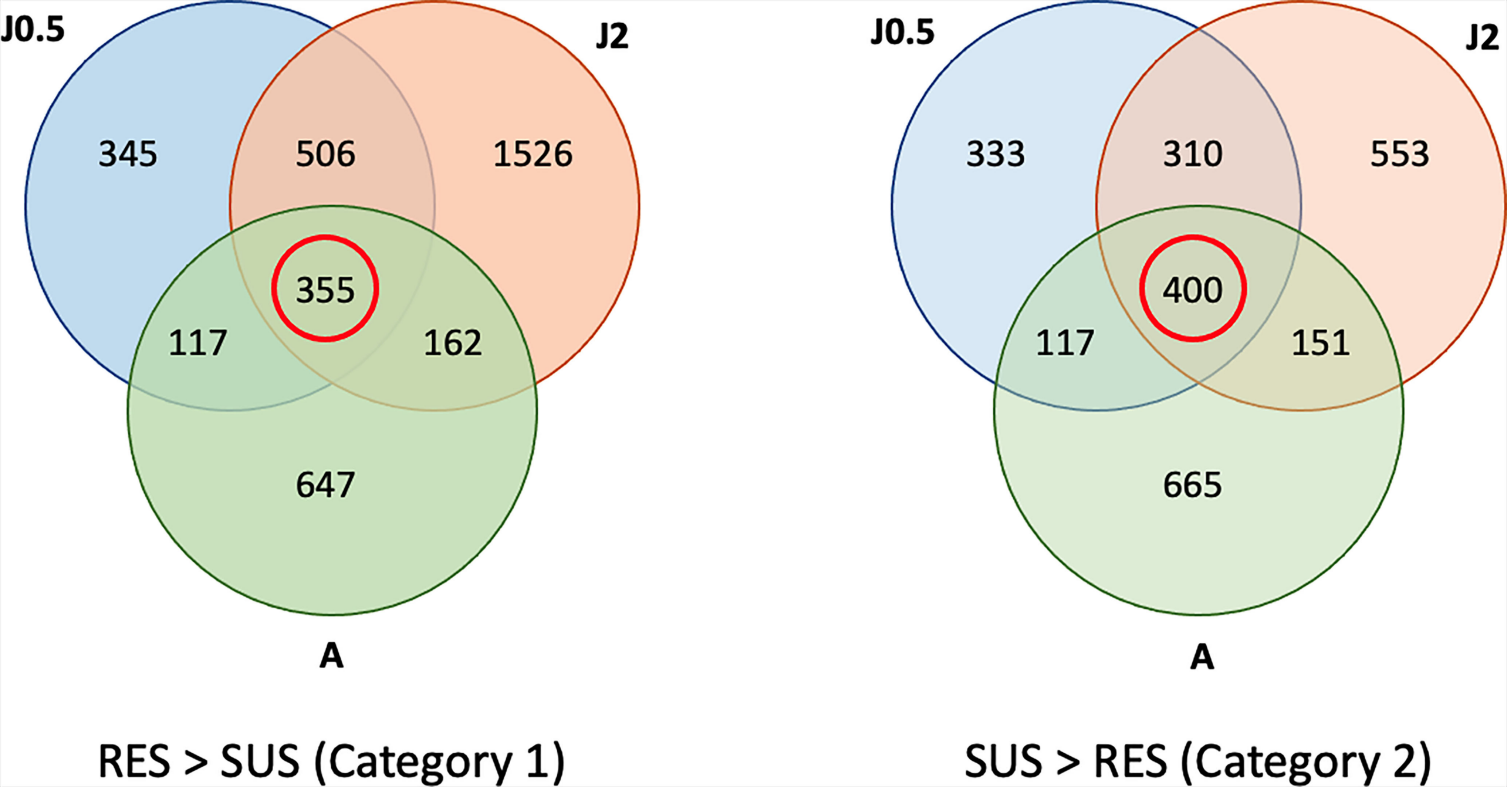
Venn diagrams showing numbers of genes in common among three comparisons of unexposed control RES and SUS snails. Contents outside of each circle refer to the specific sampling time points indicated in **Table S2** (RES vs. SUS). The three comparisons include RES vs. SUS with juvenile controls @0.5 dpe (J0.5), @2 dpe (J2), and with adult controls @40 dpe (A). The red circle shows the genes in common in all 3 comparisons, with gene transcript representation greater in RES > SUS (Category 1), or SUS > RES (Category 2). Genes in common to all three comparisons (within the red circles) were subject to further analysis.

A total of 355 genes were over-represented in common in all three RES groups (**Figure 4**) as compared to SUS and these were considered as representatives of category 1 in **Figure 1** (constitutive resistance factors). Similarly, in all 3 comparisons, 400 were over-represented in common in SUS as compared to RES and considered representatives of category 2 (constitutive susceptibility factors). The full list of transcripts placed in categories 1 and 2 is summarized in **Table S4**. Any previously reported putative resistance-associated genes identified in **Table S1** are shown in red in **Table S4**. Also, grouped by gene families are genes previously identified from Bg (or other gastropods), or genes first reported in Bg in this study.

For category 1 (constitutive resistant factors of **Figure 1**), as for all the remaining categories, there are many NA (especially non-coding RNAs) and uncharacterized features listed, a clear indication there is much yet to learn regarding the functional role of many responsive snail genes (**Table S4**). Notable in category 1 are 77 putative resistance-associated genes (**Table S1**), including 13 AIG gene family of GTPases (73), 2 FREPs/FReDs (74), cadherins, cathepsins L, cytochrome P450s, dual oxidases, ubiquitins, peroxidase, laccase-like, TNF receptor-associated factor 3, Macrophage-expressed gene 1 protein-like isoform X1 and peptidoglycan recognition protein. Mitogen-activated protein kinase kinase kinase 1-like (Bg33194), over-expressed in RES granulocytes (47) also fell into our category 1. Also of interest were sulfotransferases implicated in stress responses, a mitogen activated kinase, and an ankyrin repeat potentially involved in receptor tyrosine kinase signaling. Dentin sialophosphoprotein-like transcript, involved in mineralization, was also distinctly over-expressed in control RES snails as compared to SUS.

For category 2 (constitutive susceptibility factors of **Figure 1**), the 55 putative resistance-associated genes (**Table S1**) were noticeably different from category 1. A total of 15 lectins were identified in category 2 relative to only two in category 1. Other features of note were a thioester-containing protein, toll-like receptor 7, lipopolysaccharide binding protein, and NF-kappa B inhibitor. *FREP3.2* (Bg204) and fibrinogen-related protein K3 precursor (Bg20380) appeared in category 2 as did C-type lectin domain family 10 member A-like (Bg20382) and Multiple epidermal growth factor-like domains protein 11 (Bg24594), the latter two also found to be over-expressed in SUS hyalinocytes (47). Also of interest were genes encoding a turripepetide ici9.2 like molecule with similarity to other gastropod-produced toxins, an RNA-directed DNA polymerase, and a piwi-like protein that, based on the partial sequence we obtained (Bg31729), was homologous to Bg10170 noted by Bridger et al. (24) and subsequently implicated in retrotransposon silencing, epigenetic modification and resistance to Sm (72). We identified 3 distinct piwi genes in the BB02 genome (Bg10170, Bg28315, Bg24465), none of which were differentially expressed in either strain at any of our time points following exposure to Sm (see *Discussion*).

Genes with leucine rich repeats, pore-forming macrophage binding proteins, multiple epidermal growth factors, BTB/POZ domain-containing protein, metabotropic glutamate receptors, methyltransferases, molecules associated with core mitochondrial respiratory functions, FREPs and various proteases and protease inhibitors were noted among both Categories 1 and 2.

### Responses of SUS and RES Snails to *S. mansoni* Exposure

#### Overall Comparison of Differences Between SUS and RES Strains Exposed to *S. mansoni*

To compare the effects of exposure to Sm on gene expression in SUS and RES strains, eight different groups of exposed snails were examined, 4 for SUS and 4 for RES snails (**Table S2**), each at four time points, 0.5-, 2-, 8- and 40 dpe (see *Materials and Methods* for rationale for these time points). Responses of exposed snails were compared relative to baseline unexposed controls within the same strain at the same time (2 and 8 dpe groups used the same control snails).

**Figure 5:**
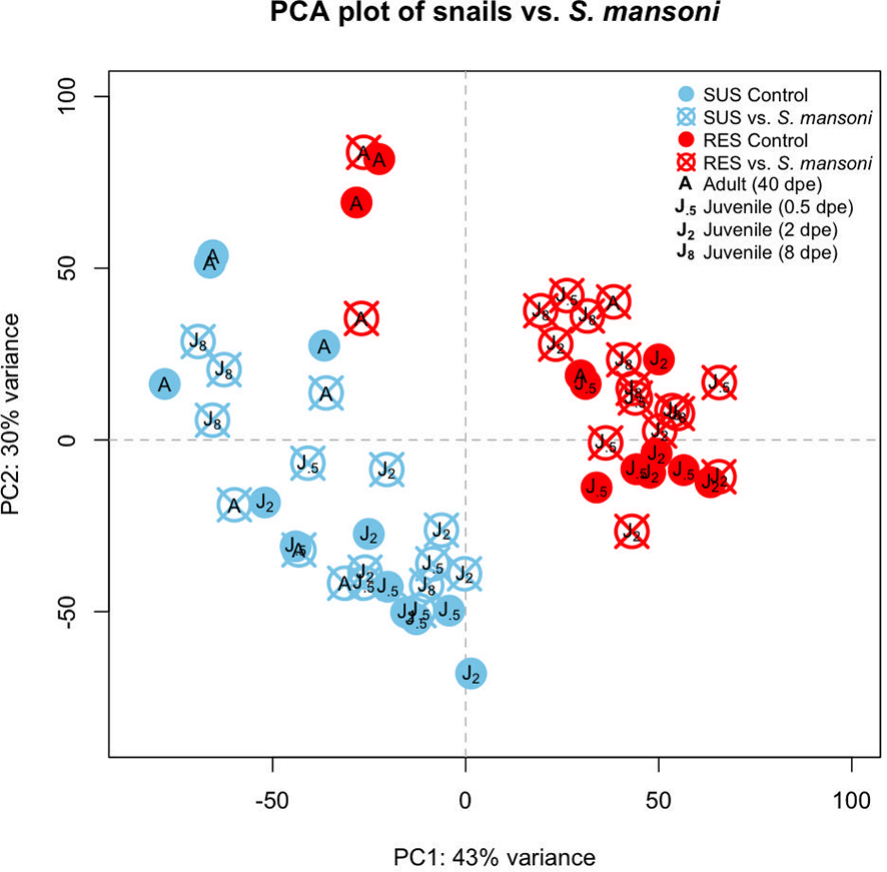
Principal components analysis (PCA) plot indicating the eight groups of snails exposed to *S. mansoni* examined in this study. Results for SUS unexposed snails are shown in light blue closed circles and snails exposed to Sm in light blue crossed circles. RES designations were similar but shown in red. “**J_.5_**” refers to juvenile snails matched in size and time to snails exposed to Sm for 0.5 days, “**J_2_**” and “**J_8_**” refer to juveniles matched to snails exposed to Sm for 2 or 8 dpe, respectively, and “**A**” refers to adult snail size and time matched to snails with patent infections of Sm (~40 dpe).

A PCA plot was generated to compare the RES and SUS responses to exposure to Sm (**Figure 5**). All Sm-exposed snails and their time-matched unexposed controls were included. In general, SUS snails tended to cluster together regardless of Sm exposure, as did RES snails. However, some RES adults (unexposed or exposed to Sm) tended to cluster more to each other than to RES juveniles. In SUS snails, there was no obvious separation between adults and juveniles.

#### Overall Differential Expression (DE) Analysis Among SUS and RES Groups Exposed to *S. mansoni*

**Figure 6:**
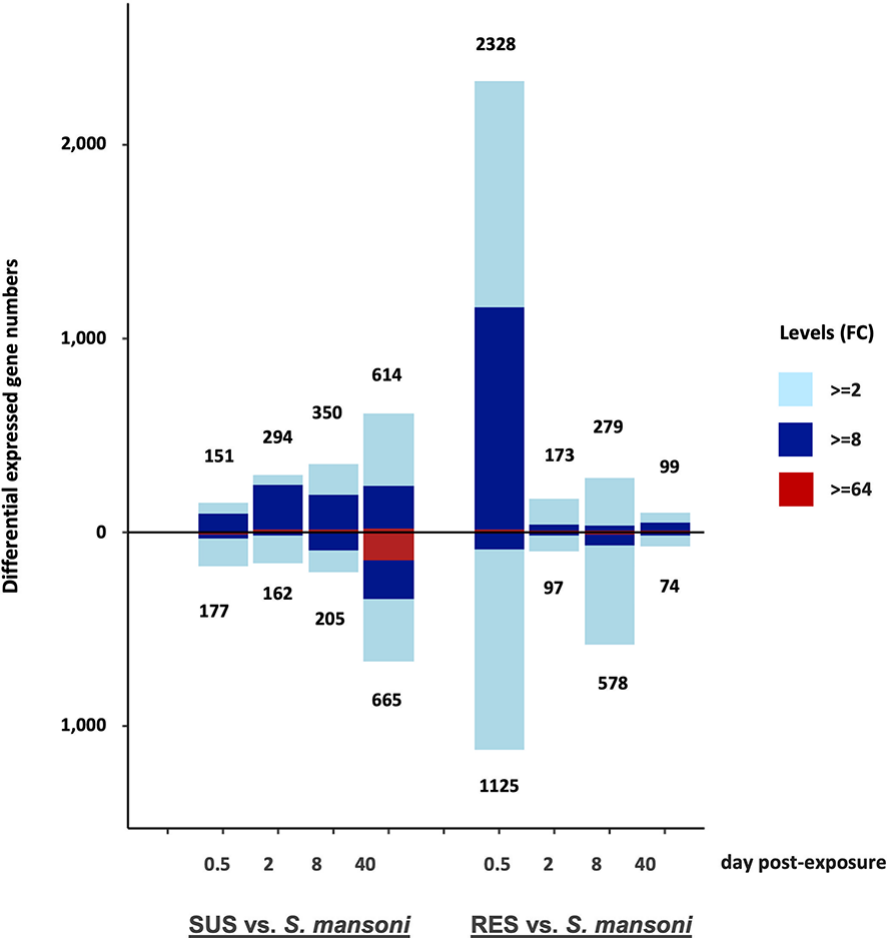
Bar graph indicating numbers of DE genes in the 8 different groups of SUS and RES snails exposed to *S. mansoni* (in **Table S2**). The number of up-regulated genes is shown above the zero line and the number of down-regulated genes is shown below the zero line. The horizontal axis shows days post-exposure for both strains. Numbers outside of each bar represent the total DE genes, either up-or down-regulated. The three colors in each bar indicate fold changes (FC) among comparisons: light blue 2-8; dark blue 8-64; and red greater than 64-fold.

A summary of the overall pattern of up- and down-regulated responses derived from the comparisons show in **Table S2** shows markedly different patterns in the responses and their time courses in SUS and RES snails (**Figure 6**). The early 0.5 dpe response of SUS snails was modest by comparison to the dramatic RES response. Thereafter, as the parasite continued to develop in SUS snails culminating with the production of cercariae by 40 dpe, the responses of SUS snails were more pronounced than for RES snails in which the parasite failed to develop. It is reasonable to expect that most parasites were eliminated by 8 dpe in RES snails, none of which shed cercariae. Also of interest is that whereas, 2, 8 and 40 dpe responses of SUS snails showed a preponderance of up-regulated genes, by 40 dpe, the presence of roughly 100 strongly down-regulated genes was of interest. For RES snails, down-regulation was most prominently noted at 8 dpe, suggestive of a reduction in the evident responses mounted earlier at 0.5 dpe.

#### Overall SUS and RES Responses Differ Over Time Following Exposure to *S. mansoni*

**Figure 7:**
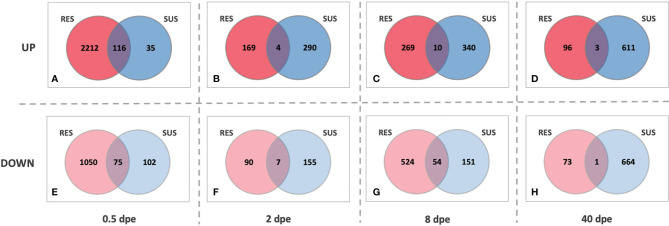
Venn diagrams of comparisons between RES (red) and SUS (blue) snails exposed to *S. mansoni* for 0.5, 2, 8 or 40-days post exposure (dpe). Indicated for both strains at the various sampling time points are genes that are either up-regulated (top panel, **A–D**) or down-regulated (bottom panel, **E–H**) relative to unexposed snails of the same strain. The numbers in the areas of overlap indicate the particular genes that respond in the same direction in both snail strains.

A different perspective on the responses of SUS and RES snails is provided by the series of Venn diagrams in **Figure 7**. At each time point, the number of genes up- or down-regulated in common to the two strains was far exceeded by the number that were uniquely differentially expressed in each strain. Also, whereas the RES snail response is clearly biased towards the early stages of exposure, the SUS response becomes much more evident as the infection proceeds, again culminating in sustained production and shedding of cercariae.

**Figure 8:**
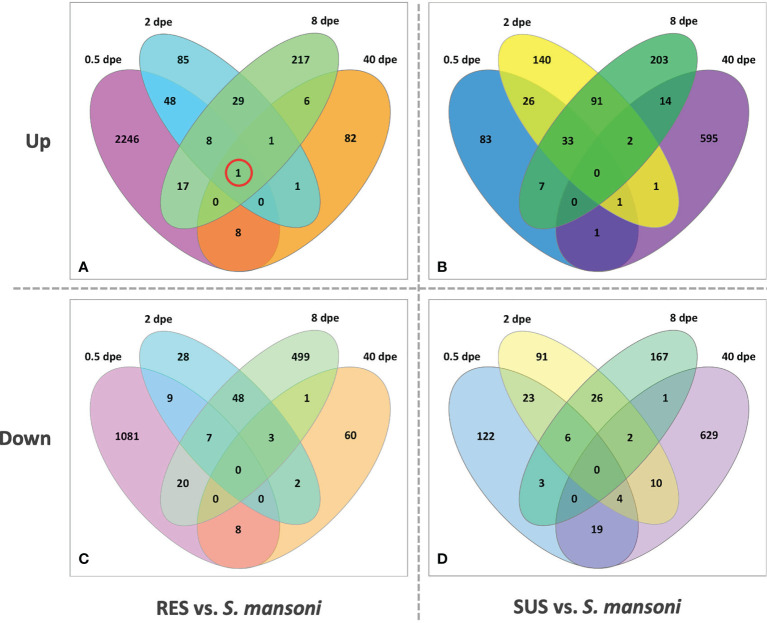
Venn diagrams of RES (left panels) and SUS (right panels) snails exposed to *S. mansoni* for 0.5, 2, 8 and 40-days post-exposure (dpe). Numbers outside each oval refer to the specific sampling time point; these sampling times are also indicated in **Table S2** (Snail vs. Sm). The upper panels **(A, C)** refer to up-regulated genes and the bottom panels **(B, D)** to down-regulated genes. The single gene circled in red in panel **(A)** (FREP 4, Bg 152) was the only one found to be consistent in its expression at all four time points.

**Figure 8** provides for both RES and SUS a view of how the response changes over time. The early RES up-regulated response is followed by a notable down regulated response by 8 dpe. Thereafter, in the absence of a persisting parasite, the response of previously exposed RES snails is unremarkable. It is also noteworthy that one gene, FREP4 (Bg152), is consistently up-regulated during infection in RES strain (**Figure 8A**, circled). In contrast, in SUS snails, there tends to be a preponderance of uniquely expressed genes either up- or down-regulated at each time point, especially so at 40 dpe. This suggests the Sm-infected snails are responding in a more continuous and dynamic way to the presence of living parasites, and the mixture of up- and down-regulated genes is suggestive of a complex interaction, one that could involve elements of up-regulated host susceptibility, stress or tolerance responses, and down-regulation of host defense or reproductive functions, or perhaps snail genes targeted by parasite manipulations (see listing of categories below).

### Categories of Responses of RES and SUS Following Exposure to *S. mansoni*

#### Category 3 – Stress-Features up-Regulated in Both SUS and RES at 0.5 or 2 dpe

As shown in **Table S5**, a total of 117 genes were up-regulation in both strains at 0.5 dpe, whereas only 5 were up-regulated in common at 2 dpe. Numbers of putative resistance factors identified in **Table S1** for the two time points were 11 and 1, respectively. Although we considered that up-regulated genes in snails of both strains might be indicators of a generalized stress response upon exposure to a parasite, the number of putative resistance factors was lower for this category than the other six categories. Also, features often associated with a stress response like heat shock proteins or cytochrome P450s were not represented in category 3, but the stress-associated gene chromobox protein homolog 1 (BgCBx1) (72) was up-regulated in both SUS and RES snails at 0.5 dpe. The most noteworthy components in this category were several genes that had some relationship to ubiquination or subsequent protein degradation (kelch-like proteins, baculovirus IAP repeat-containing protein 3-like, E3 ubiquitin-protein ligase RNF4-like, nmrA-like family domain containing protein-1 like, universal stress protein MSME G9950, and tetratricopeptide repeat protein 25-like). Ubiquination may play a role in marking some parasites or their products for degradation. Ficolin 2-like and a toll-like gene indicative of pattern recognition associated with innate immune responses were also found in category 3. Few genes in category 3 were elevated to a log2FC >5.

#### Category 4 – Resistance Factors Up-Regulated in RES Snails at 0.5 and 2 dpe to *S. mansoni*

A total of 2,212 genes were significantly elevated in expression at 0.5 dpe (**Table S6**), the highest number reported for any treatment (**Figure 6**), the vast majority of which has log2FC values <5. That number dropped to 170 genes at 2 dpe, of which only 57 were up-regulated in common to both time points. Notable among the genes expressed at 0.5 dpe were 18 general stress response genes (among them glutathione-S-transferases, heat shock proteins 12.2 and 70, universal stress protein SAS1637, stress response protein NST1-like and alpha-crystallin B chain-like) and 34 genes identified in **Table S1** as putative resistance candidates, most notably 18 different FREPs/FReDs. Additionally, 29 different lectins (20 uniquely expressed at this time point) were up-regulated. We were also surprised to observe that 48 different G protein-coupled receptors (GPCRs) were up-regulated; GPCRs were affected much more for this treatment and time point than for any of the others we examined.

The 2 dpe response was quite different with only one stress-related gene up-regulated (glutathione S-transferase) as were 61 of the putative resistance factors identified in **Table S1**, 21 of which were FREPs/FReDs. Among the up-regulated genes were 15 lectins (6 unique to this time point) and 7 GPCRs. Of the 57 genes identified from both time points, only glutathione S-transferase was identified as a general stress-related feature and 11 were previously identified putative resistance factors, including 7 lectins. Ten genes in category 4 were elevated to a log2FC >5, all at 2 dpe, including 3 FREPs/FReDs, a galectin, and two C-type lectins.

#### Category 5 – Factors, Including Putative Resistance Factors, Down-Regulated in SUS Snails at 0.5 and 2 dpe to *S. mansoni*

A total of 178 genes were significantly down-regulated in SUS snails at 0.5 dpe (**Table S7**), most having modest levels of down-regulation. Amongst them were 5 stress-related genes, 29 putative resistance factors (**Table S1**) and 5 lectins. Several down-regulated genes were epithelium-protective mucins or involved in cell-cell adherence or cell attachment. Among the more noteworthy putative resistance factors down-regulated were three members of the AIG/GIMAP family, a nuclear factor NF-kappa B p100 subunit-like isoform transcription factor and toll-like receptors 7 and 8. FREP4 was significantly down-regulated at 0.5 dpe. Also of note and of potential relevance were genes that might influence neurotransmission like putative copper-containing amine oxidases, a DBH-like monooxygenase protein 1 homolog, 3 GPCRs, a prolactin-releasing peptide-like and a metabotropic glutamate receptors 2-like molecule. The significance of these latter molecules may lie more in parasite effects on host reproduction then immune responsiveness.

At 2 dpe, 164 genes were down-regulated of which one stress-related gene, 16 putative resistance-associated genes and 4 lectins were detected. Perhaps the most significant gene detected was macrophage expressed gene 1 protein-like, a perforin-like molecule. Also of interest was laccase-2-like, a likely phenoloxidase-encoding gene with a possible role in cross-linking phenolic compounds and in melanization. As for genes down-regulated at both 0.5 and 2 dpe, only 34 were found of which 6 were putative resistance factors (**Table S1**), suggestive of a rapid change in any *S. mansoni*-induced effects in early-stage infections. In general, values of log2FC < -5 for commonly expressed transcripts were rare among category 5 transcripts.

#### Category 6 – Factors Down-Regulated in SUS Snails at 8 and 40 dpe to *S. mansoni*, Potentially Indicative of Parasite Inhibition or Manipulation

At 8 dpe, 206 genes were down-regulated (**Table S8**), 6 of which were stress-related factors and 12 were putative resistance factors mentioned in **Table S1**. Additionally, 7 lectins (including Bg selectin, hemocytin-like, macrophage mannose receptor-1-like and perlucin among them) were also down-regulated as was again the putative perforin, macrophage expressed gene 1 protein-like. Two additional genes of note were 5-hydroxytryptamine receptor 2-like (a serotonin receptor) and sialate O-acetylesterase-like (required for maintenance of tolerance in vertebrates by down-regulating lymphocyte antigen receptor signaling). The degree of down-regulation was modest as compared to 40 dpe samples.

At 40 dpe, 666 genes were down-regulated, many with values of log2FC <-5 indicative of strong suppression, including 5 identified as stress-related genes and 36 putative resistance genes (**Table S1**). Remarkably, 20 different genes identified were lectins or had lectin-like domains (4 of which were among the 36 previously identified resistance-associated factors), 19 of which were differentially expressed uniquely in this category. Along with lectins, several other prominent categories of molecules were down-regulated including 14 GPCRs, glycosyl transferases, digestive enzymes, extracellular matrix components, mucins, reproduction-related genes, and 3 aerolysin-like and 1 physalysin pore forming molecules with homology to biomphalysin gene family (87). Several uncharacterized proteins with log2FC values < -8 were noted, indicating there is much to learn regarding the snail proteins affected or targeted during Sm infection. The overall profiles of 8 and 40 dpe were strikingly different as only 3 genes were down-regulated in common to both time points.

#### Category 7 – Factors Up-Regulated in SUS Snails at 8 and 40 dpe to *S. mansoni*, Potentially Indicative of Tolerance or Host Protection Responses

At 8 dpe, 351 genes were up-regulated (**Table S9**) which included 7 general stress factors, 28 putative resistance factors from **Table S1**, 2 additional FREPs/FReDs, 6 lectins and 11 GPCRs. At this time point, most genes had a log2FC value <5 and the response was not particularly striking.

By 40 dpe, the response was more dramatic, with 615 genes up-regulated, which included 10 general stress factors, 72 putative resistance factors (more than for any other time point or treatment), 4 additional FREPs/FReDs, 33 lectins (28 uniquely differentially expressed only at this time point) and 11 GPCRs. Several genes had log2FC value >5. Prominent among the genes noted were antimicrobial factors (bactericidal permeability increasing protein, peptidoglycan recognition protein, beta 1-3 glucan binding protein, lysozyme, macrophage mannose receptor 1 like), TLRs 2 and 8, TNF receptor-associated factor 5-like probably involved in signal transduction, venom-like proteins (turripeptide lci9.2-like, U3 aranetoxin-Ce1a-like), and several factors likely involved in reproduction (temptin, yolk-ferritin, FMRFamide receptor-like, neuropeptide Y receptor type 5-like). As with category 6, the overall profiles at 8 and 40 dpe were strikingly different, and only 17 genes were up-regulated in common to both time points.

#### Groups of Genes Not Covered by Our Seven Categories

Our categories 1-7 excluded certain groups of genes such as those uniquely up-regulated in SUS snails at 0.5 and 2 dpe, and those down-regulated in RES snails at 0.5, 2, 8 and 40 dpe. An overview of these groups is provided in **Tables S10**, **S11**. In general, most differentially expressed genes had modest levels of up- or down-regulation (depending on the group), and putative resistance-related factors were not prominently represented. The uniquely up-regulated response of SUS snails at 0.5 dpe involved few genes, in sharp contrast to the response of RES snails at the same time, suggesting SUS snails simply did not detect the presence of Sm, or their responses had been successfully suppressed by the parasite. At 2 dpe, up-regulated genes known to dampen signaling responses in other systems (e.g. mitogen-activated protein kinase binding protein 1) or involved in ubiquination were noted. Several down-regulated genes were seen in RES snails, especially at 0.5 dpe, including putative defense-related molecules like lectins, along with mucins, cadherins, collagen, actins, dyneins, hemoglobin, and extracellular matrix components. At 2 dpe, some genes indicative of protections against reactive oxygen species like glutathione-S-transferases were down-regulated and by 8 dpe, when several genes were down-regulated, prominent among them were AIG/GIMAPs and several genes involved in intracellular lysosomal catabolism. The latter is potentially indicative of the waning presence of parasites which would have been largely dismembered by phagocytosis by that time point. The down-regulation RES response at 40 dpe was modest and did not have any obvious signatures, perhaps not surprising given the absence of viable Sm from such snails.

### *B. glabrata* lectins and GPCRs: Remarkable Specificity in Their Response Profiles

**Figure 9:**
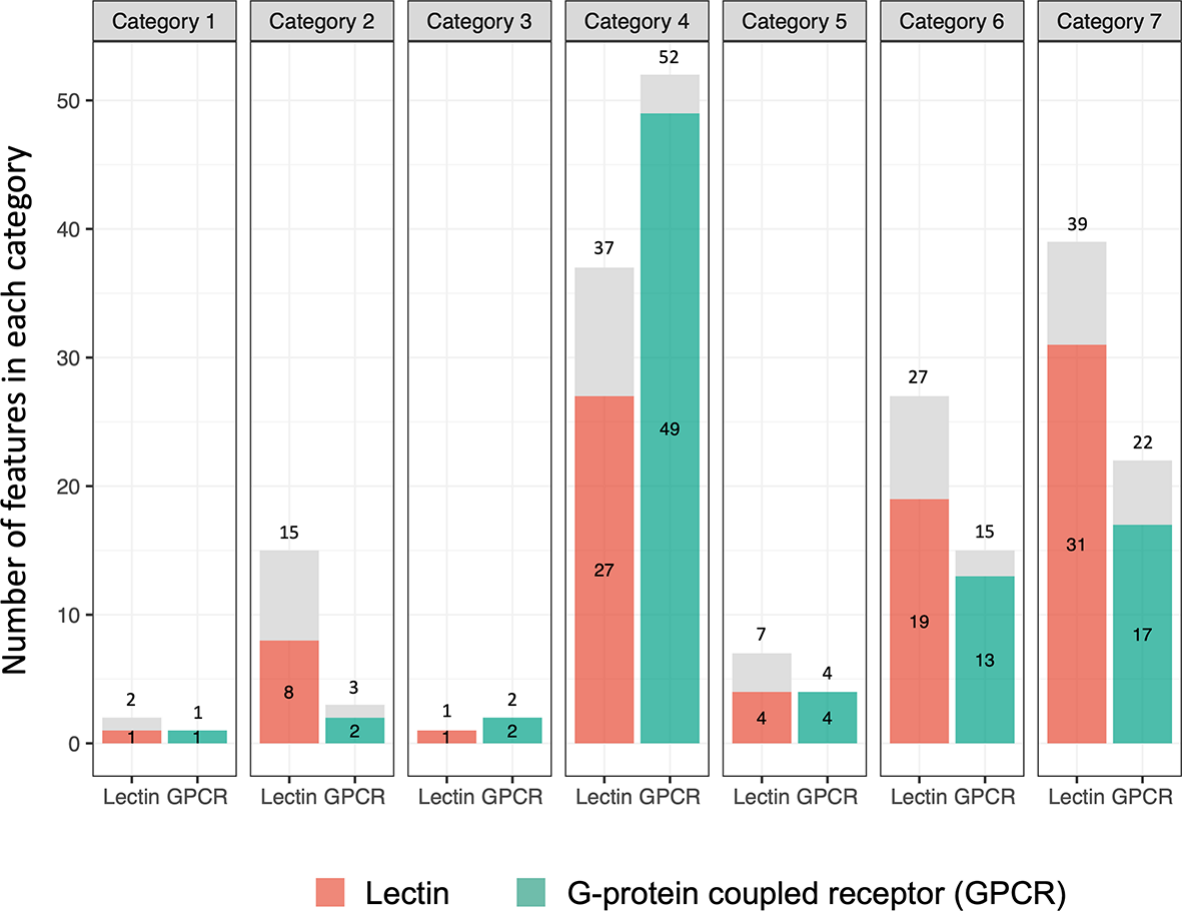
Bar graph showing numbers of lectin and G-protein coupled receptor (GPCR) genes in the 7 categories. The number outside of each bar is the total number of genes observed. The number inside the bar is the number of genes seen unique to that category (highlighted in orange or green boxes).

As shown in **Figure 9** (**Tables S12**, **S13**), one of the most noteworthy features of the *B. glabrata* transcriptome relative to exposure to Sm was the considerable extent to which the responses of the 111 putative lectins and 91 GPCRs noted were exclusive to one of the 7 different categories we identified. The prominence of GPCR involvement was surprising. Expression of 91 of the 111 (82.0%) lectins and 86 of 91 (94.5%) GPCRs were restricted to a single category. Especially noteworthy in this regard were categories 4, 6 and 7. For category 4, 27 of 37 (73.0%) lectins and 49 of 52 (94.2%) GPCRs detected were uniquely differentially expressed only in this category. Categories 6 and 7 suggest that one group of lectins or GPCRs is uniquely marked for down regulation (category 6) and another group of lectins and GPCRs is uniquely up-regulated (category 7), indicative of very different functional roles for these two groups of lectins in the complex environment presented by the shedding snail. In contrast, the numbers for AIG/GIMAPs, FREPs/FReDs, or other genes identified by genome-wide mapping studies were less likely with respect to expression changes to be confined to a specific category.

### An Overview of Transcriptomics Profiles for Putative Resistance Candidates Identified From Previous Genome-Wide Mapping Studies (GWMS), or From Studies Focused on Particular Groups of Molecules

In this section, relative to the 7 response categories we delineated, we examine the genes identified by available GWMS studies of resistance-associated factors in Bg, and by groups of Bg genes identified by other means such as their transcriptional or protein responses, designated here as feature-specific studies (**Tables S3**, **S14**). Most of the genes identified by the GWMS studies were found in our database (>96%). About a third of all the genes in GWMS and the feature-specific studies were not found to be differentially expressed. For both GWMS and feature-specific studies, more genes were seen in category 1 than category 2, although this varied somewhat from study to study. This imbalance was especially notable for Thioester-containing proteins (TEPs) and AIG/GIMAPs.

For the four GWMS studies, of 55 genes identified, about half (29, or 52.7%) were responsive to Sm, slightly more in SUS (18) than RES (16). For feature-specific studies, about 41% (111) were responsive to Sm, with more (83) responsive in RES than SUS (43). For the 4 GWMS studies, most of the Sm responsive genes in RES snails were detected at 0.5 or 2 dpe (93.8%), whereas most of the Sm responsive genes in SUS snails were responsive at 8 or especially 40 dpe (72.2%). For the feature-driven studies, the same pattern was noted: in RES snails most of the Sm responsive genes were detected at 0.5 or 2 dpe (65.1%), whereas in SUS snails most of the Sm responsive genes were seen at 8 or especially 40 dpe (79.1%). In general, AIG/GIMAPs and TEPs were not particularly responsive to Sm exposure.

With respect to representation of GWMS or feature-specific studies (**Table S3**) in our remaining five categories, category 3 was not well-represented, just as it was not in our overall consideration of all genes. Category 4 had the most Sm-responsive genes, with the largest group of molecules in this category being FREPs/FReDs. Like category 3, category 5 was not particularly prominent, suggestive perhaps a lack of recognition of Sm in SUS snails rather than a large measure of overt parasite manipulation. The number of genes down-regulated in shedding snails (category 6) was similar to the number up-regulated at the same time (category 7). For category 6, it is not surprising that the Hathaway et al. (2010) study (88) revealed proportionately more responders than other studies since egg mass components were targeted by this study, and egg mass production is known to be down-regulated in shedding snails which are typically castrated. The GWMS studies identified more genes in category 7 than category 6.

### Uncharacterized Proteins and Features With No Annotation (NA)

It should not be overlooked that 30~40% of the transcripts we identified were categorized as “uncharacterized protein” and “NA” even after multiple blasts to different databases. The literature on genes in these two categories is limited for *Biomphalaria*, one study focusing on transcripts of *Biomphalaria pfeifferi* (89) and another on a proteomics analysis of Bg plasma (90). We blasted the uncharacterized proteins from these two studies in search of homologous sequences and the associated VB ID, then compared expression responses. We found homologs of at least 4 transcripts in the *B. pfeifferi* study (89) that were up-regulated in RES snails at 0.5, 2 or 8 dpe in our study. Considering we noted that several uncharacterized proteins and NA features were differentially expressed to a strong degree, it is likely many play key roles in snail-schistosome interactions and are deserving of further characterization.

### Co-Expression Network Analysis for RES Snails Exposed to *S. mansoni*

**Figure 10:**
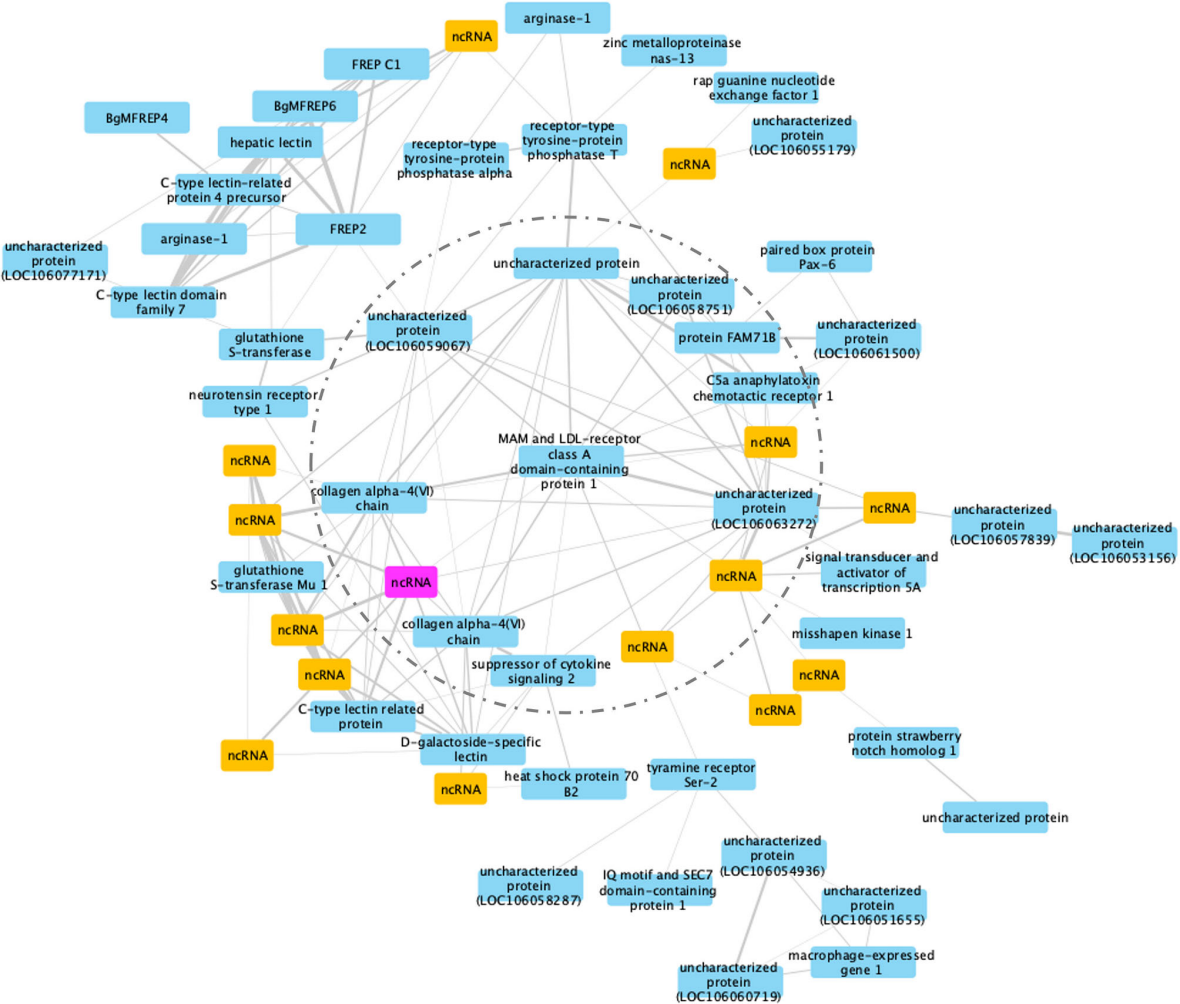
A co-expression network in module #19 of RES strain with resistant-related genes. Protein coding genes including annotated and uncharacterized proteins are in blue boxes; non-coding RNA (ncRNA) genes are in yellow boxes. The ncRNA appearing in both RES and SUS networks was highlighted in pink.

Co-expression modules are defined as groups of genes that share similar expression patterns, and although they do not necessarily represent a particular biochemical pathway, they do tend to be co-regulated and functionally related. For RES snails, 45 gene modules were identified (**Figure S2**). Modules #6 and #19 had similar high significant correlation coefficients (Pearson correlation coefficient R^2^ = 0.71, *P* = 2 x 10^-5^ for module #6, and R^2^ = 0.70, *P* = 3 x 10^-5^ for module #19). Among the sub-networks present in one module, the largest usually accounts for 90% of the nodes, so only the largest network was considered further. Module #19 was chosen for further analysis because of the larger number of DE genes (61) in the network (**Figure 10**).

MAM and LDL-receptor class A domain-containing protein 1-like (Bg17695) was a hub gene, a gene co-expressed with high number of other genes in the co-expression network including those in an inner circle of genes (dash line circled, **Figure 10**) with slightly less connectivity, and outer layers of genes with only one connection (co-expression correlation) to the inner layer genes. Variants of this particular hub gene have been found in a population of the bivalve mollusc *Mercenaria mercenaria* exhibiting some resistance to an uncharacterized eukaryotic parasite called QPX, or Quahog Parasite Unknown (91).

Several additional genes with immunological functions were found on this network: six immunoglobulin and lectin domain containing molecules (VIgLs) including 4 FREPs/FReDs (FREP2; BgMFREP4, BgMFREP6 and FREP C1) and 2 C-type lectin related proteins (CREPs). Most of the genes in the network responded early following Sm exposure (0.5 and 2 dpe) (**Table S15**), including: arginase-1, collagen alpha-4(VI) chain, glutathione S-transferase, heat shock protein 70 B2, and macrophage-expressed gene 1. Several (15 of 61, or 24.6%) genes in this network are ncRNAs. Although we know little about the involvement of ncRNA in the immune responses in RES snails to Sm exposure, the role ncRNAs play in complementary binding to mRNAs clearly deserve more investigation.

### Co-Expression Analysis in SUS Snails Exposed to Sm

**Figure 11:**
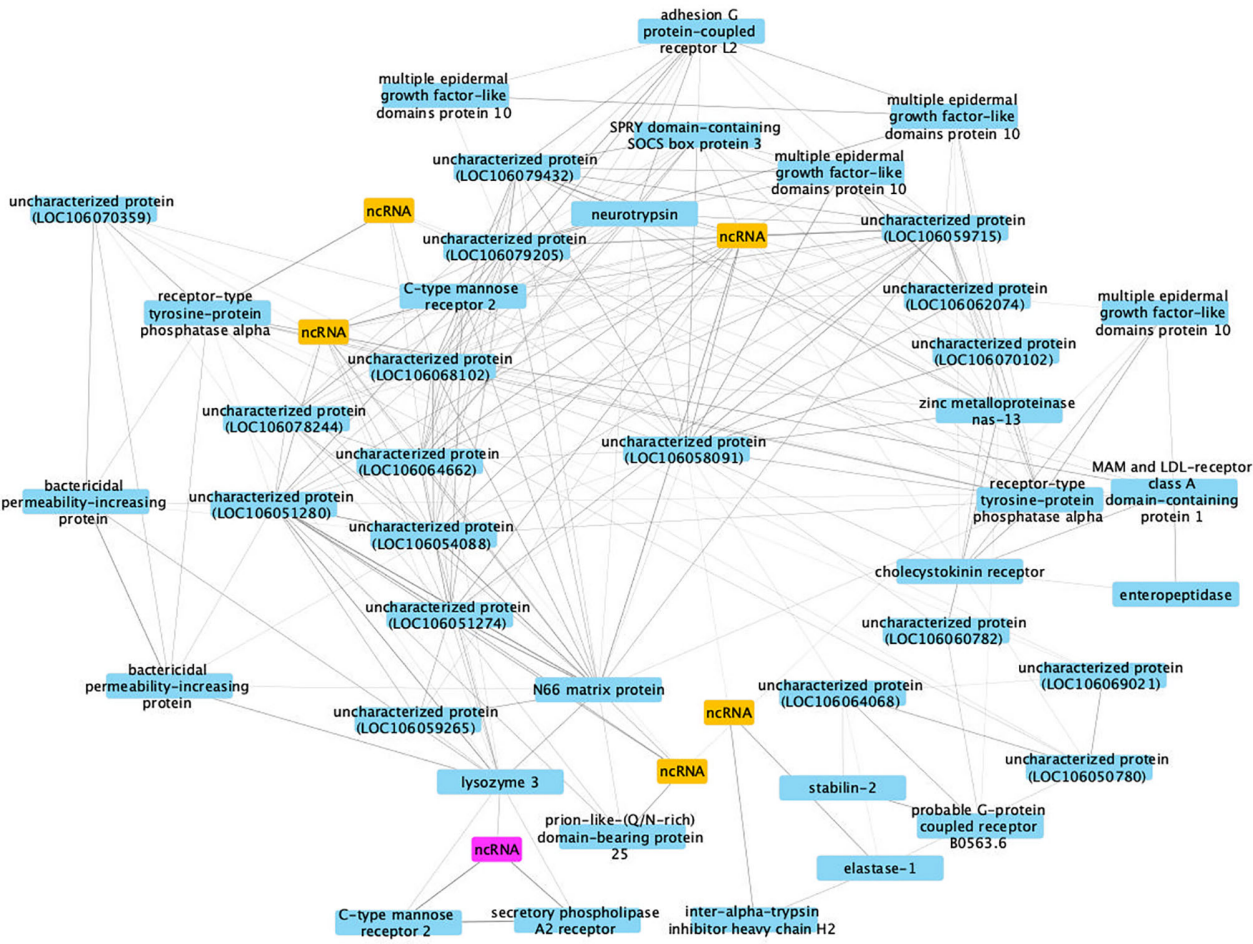
A co-expression network in module #4 of SUS strain with infection-related genes. Protein coding genes including annotated and uncharacterized proteins are in blue boxes; non-coding RNA (ncRNA) genes are in yellow boxes. The ncRNA appearing in both RES and SUS networks was highlighted in pink.

This co-expression analysis generated 29 separable modules, and module #4 had the most significant correlation coefficient (Pearson correlation coefficient R^2^ = 0.93, *P* = 2 x 10^-5^, **Figure S3**). Due to the large numbers of nodes (224 genes) identified in the most important network in module #4, only differentially expressed genes with co-expression weight greater than 40% were kept for further analysis (**Figure 11**).

The hub gene is an uncharacterized protein (LOC106058091, Bg29733), based on lack of BLAST hits to the NCBI non-redundant protein/nucleotide databases. However, domain prediction indicates its similarity to a CTX-related type I transmembrane protein (PTHR12231) believed to be involved in thymocyte selection in *Xenopus* (92). The layer of genes arrayed around the hub also show a high connectivity to each other and nearby genes, indicating their importance in the network (**Table S15**). Although many components of the network encode proteins of uncharacterized functions or are ncRNAs, there are also several annotated genes up-regulated in 40 dpe shedding snails, including adhesion GPCR L2, bactericidal permeability-increasing protein, C-type mannose receptor 2, multiple epidermal growth factor-like domains protein 10 and another PTP, receptor-type tyrosine-protein phosphatase alpha (see RES network also). These genes could play critical roles in improving anti-bacterial immune response in SUS snails shedding cercariae and thus subjected to repeated epithelial breaches and overall physiological stress.

Interestingly, a ncRNA (Bg20159) was identified in both RES and SUS networks (highlighted in pink in **Figures 10**, **11**) and was up-regulated in both RES snails at 0.5 dpe (log2FC=4) and in SUS snails at 40 dpe (log2FC=5.77). This ncRNA may play a critical regulatory role in immune responses in Bg and deserves further investigation.

Protein coding genes including annotated and uncharacterized proteins are in blue boxes; non-coding RNA (ncRNA) genes are in yellow boxes. The ncRNA appearing in both RES and SUS networks was highlighted in pink.

## Discussion

Comprehensive transcriptomics approaches like RNA-Seq offer a distinctive window into the responses of hosts to parasites, and when the responses of hosts that are susceptible or resistant to infection can be compared, then we stand to gain new insights as to how resistance might be achieved. It is important to offer several caveats regarding transcriptomics in general and our approach in particular. Our study takes a whole-organism approach so might overlook rare transcripts or have the insights offered by individual organs or even cells diluted in the whole-body response. Also, many of the responding genes have not been annotated or their functions in molluscs are poorly known, so we are far from having a full list of all the relevant players involved in host defense. A change in transcription activity might be part of an independent host process like sexual maturation or might be vulnerable to erroneous interpretation (an up-regulated gene might function as an unknown inhibitor of an immune response, for example). Some genes, although their expression is not altered by exposure to infection, may still play important roles in determining resistance status. Nonetheless, our RNA-Seq approach does help to focus attention on genes likely to play a role in defense, and offers a more comprehensive framework for further consideration, including laying the groundwork for awaited functional studies aimed at validating the actual impacts of certain host genes on parasite well-being. We also note this study dwells only on host features. Sm sequences have been excluded from the current analysis and will be investigated separately.

We have focused on 7 different categories of snail responses in attempt to direct attention to what we feel are the most interesting aspects of the responses. These categories are devices to help make sense of the many gene responses noted. Whereas, given the methods we used, we are confident that this binning process has captured the immune-related genes whose expression profiles fit the designated category, this is not to say that *all* genes we found with such an expression profile should necessarily be considered an immune-related gene. The expression profiles of some genes might match the category designation just by chance. We also acknowledge that the boundaries between categories may be somewhat arbitrary, but argue they nonetheless provide a convenient way to organize the data. We feel this approach has been useful, including in enabling us to tease out distinctive category-specific responses in genes like lectins and GPCRs.

Whereas roles for lectins in invertebrate immune responses have long been recognized (89), the potential role played by GPCRs in sensing “stranger” or “danger” signals in invertebrates, including molluscs, is only just beginning to be appreciated (93, 94). GPCRs belong to a large, evolutionarily related group of signaling proteins now well-appreciated for their role in mediating responses to visual or olfactory stimuli, hormones, neurotransmitters, metabolites and cytokines, and for playing important roles in regulating vertebrate immune responses (95). A total of 241 GPCR-like genes have been identified in the BB02 *B. glabrata* genome (12). Although GPCRs are obviously involved in several processes, the fact that the largest repertoire of responsive GPCRs noted in this study occurred in category 4 snails undergoing active resistance responses (52 different GPCR genes up-regulated, 49 unique to this category) is certainly suggestive of a GPCR role in defense against schistosome infection. Likewise, large specific repertoires of GPCRs either down-regulated (category 6) or up-regulated (category 7) in snails actively producing cercariae suggest snail GPCRs play important roles in influencing schistosome-snail interactions.

For category 1, constitutive resistance factors, we looked in both unexposed juvenile and adult control snails for those genes that are inherently more represented in RES than SUS snails. For category 2, constitutive susceptibility factors, we identified genes conversely more commonly expressed in unexposed control SUS snails. There were many genes in common between juvenile and adult snails within a strain in the extent to which they were over- or under-represented relative to the other strain. In general, from both the relevant previous literature (**Table S1**) and from our comparison of GWMS and feature-specific studies (**Tables S3**, **S14**), the number of putative immune or resistance related factors in category 1 exceeds the number found in category 2. A recently published transcriptomics study of single granulocytes or hyalinocytes from unexposed snails of the same two stains we studied showed a similar broad pattern: expression differences between the two cell types were overridden by strain differences, and in general, RES hemocytes were “more immunologically prepared” than were hemocytes from SUS snails (47). Whereas we found AIG/GIMAPs (73) and cytochrome p450s were highlights of category 1, category 2 revealed an over-representation of particular lectins, over half of which were not subsequently responsive in any of the Sm-exposed snails. The homolog of *FREP3.2* over-expressed in Category 2 snails is of interest because this particular *FREP* has been implicated in resistance responses, however it is known that SUS than RES snails have different allelic forms of this gene (74). Whereas the single cell transcriptome study (47) noted a pattern for higher expression of some FREPs, TEPS and biomphalysins in RES than SUS resting hemocytes lacking immune stimulation, we found strain differences to become more accentuated following exposure to Sm (see below).

In general, for genes we found showing a constitutive inter-strain bias, their response to Sm exposure was variable: some were not highly responsive to Sm exposure, but this by no means should preclude them from further consideration for playing important roles in this host-parasite interaction. The strongest responses to Sm exposure we saw generally did not involve members of category 1 and 2.

For category 3, we reasoned there might be a subset of snail genes responsive to exposure to a parasite (here viewed as the possible equivalent of a needle stick) that might signal a generalized, all-purpose stress response, regardless of the Sm-susceptibility status of the snails. Consequently, we identified genes that were up-regulated in both SUS and RES snails shortly following exposure, at 0.5 and 2 dpe. We excluded later time points from this category thinking the disparities in extent of parasite development (with attendant increased parasite manipulation and pathogenesis) would swamp out a more generalized stress response. Although we did not observe many classic elements of a generalized stress response in category 3, we did notice the chromobox protein homolog 1 (*BgCBx1*) to be up-regulated in both SUS and RES snails at 0.5 dpe, a Bg gene recently identified by Smith et al. as part of an early (0.5 to 2 *hours* post-exposure) stress response relating to resistance (72). This gene is associated with epigenetic gene repression. It is possible more generalized stress genes would have been identified if we had sampled earlier points post-exposure. However, because we did see some of the classic elements of a generalized stress response in the RES snails of category 4 (see below), it is possible that Sm either avoids detection or actively suppresses stress-related or other responses in SUS snails at 0.5 and 2 dpe. Our evidence favors the first interpretation as we saw no convincing widespread evidence of strong down-regulation of stress or immune/resistance genes in SUS snails during the same time points (see category 5 below).

Category 4 delimited the genes up-regulated at 0.5 and 2 dpe in RES snails and as such, might be expected to contain the particular genes long sought as essential to resistance. Encapsulation and destruction of recently penetrated Sm miracida/mother sporocysts is well underway in this time frame in our RES snails (38). Our results indicate a complex, multi-faceted and highly distinctive response is initiated within 12 hours of exposure in RES snails, with more up-regulated genes (2,212) than for any other treatment or time point in our study. The pronounced FREP/FReD, lectin and GPCRs responses are all noteworthy. The latter two groups of features, although known from Bg (12, 96), were surprising in their responses and clearly require further investigation because: 1) so many representatives of each were engaged at this early time point; 2) for most their altered expression was noted only at this time point; and 3) they were differentially expressed only in RES snails. By 2 dpe, although the RES response was diminished in terms of numbers of responsive genes, it revealed higher levels of expression for many genes than noted at 0.5 dpe, suggestive of a more developed, later phase in the response to infection. Obviously, inclusion of more time points to track the RES response would be desirable, including the 0-4 hour period recently discussed by Smith et al. (72). In this latter study, expression of one of the three *Bgpiwi* genes was associated with Sm resistance in BS-90 snails and with suppression of expression of the reverse transcriptase-encoding sequence of the non-LTR retrotransposons (72, 97) *nimbus*. In contrast, *nimbus* expression in the absence of *piwi* was associated with susceptibility to Sm. In the timeframe of our study beginning at 12hpe, we did not see evidence of differential expression of either *piwi* or *nimbus* in either RES or SUS snails. Awaiting further study is to learn if and how the complex response we noted at 12hpe in RES snails is connected to the genomic changes proposed by Smith et al. (72) occurring within a few hours of exposure to Sm.

Category 5 included genes down-regulated in susceptible snails at 0.5 and 2 dpe, potentially representing putative resistance/defense factors targeted by Sm. Included were 29 factors previously associated with resistance. FREP4 was notable for being significantly down-regulated at 0.5 dpe, in contrast to its persistent over-expression in RES snails following exposure to Sm. This gene is known to have allelic differences between SUS and RES snails (74). The extent of down-regulation for genes in this category was generally modest however, not approaching the levels seen later in infection of SUS snails. As noted above for category 3 responses, the pattern suggests Sm does not provoke early strong across-the-board down-regulation of immune genes in SUS snails. Any such effects might be hard to detect in snails at 0.5 or 2 dpe though because the biomass of Sm in snails at these times is much smaller than at 8 dpe, or especially at 40 dpe.

Category 6 also includes genes down-regulated in SUS snails, but at 8 and 40 dpe, with the thought that more advanced parasite development might be more revealing with respect to parasite-mediated inhibition or manipulation of host biology. The degree of down-regulation (log2FC values <-8 noted for several genes) at 40 dpe was the highest noted for any of the time points in this study. Among the conspicuous genes down-regulated at 40 dpe were 36 previous identified putative resistance factors, 20 lectins, 14 GPCRs and putative perforins. The responsive lectins and GPCRs were again remarkable in that most were altered in expression only in this category, again suggestive of a specific strain and time dependence for their altered (in this case diminished) expression.

Category 7 featuring up-regulated responses in SUS snails at 8 or 40 dpe, was considered to be indicative of possible tolerance or protective responses mounted by the snail to minimize the impact of parasitism at a time when the parasite is placing heavy demands on its host (76). More putative immune factors (69 from **Table S1**) were up-regulated in snails actively shedding cercariae (40 dpe) than for any other time or treatment in our study. Prominent lectin, GPCRs and antimicrobial responses were again noted, consistent with the notion of increased protection of the host from secondary infections. The majority of the responsive lectins and GPCRs up-regulated were again unique to this category.

The network analysis study for both RES and SUS snails identified several genes with a significantly correlated pattern of expression following exposure to Sm. Not surprisingly, the networks identified for SUS and RES were quite different with few overlapping genes. The RES network accentuated the response occurring at 0.5 and 2 dpe, a time that genes rejection of the parasite, and includes a number of genes previously implicated in their involvement in resistance such as FREPs/FReDs, glutathione S-transferase and macrophage expressed protein 1. Noteworthy too is that the hub gene, MAM and LDL-receptor class A domain-containing protein 1-like and two other prominent network genes, both tyrosine phosphatases (PTPs receptor-type tyrosine-protein phosphatases) resembled genes from the bivalve *Mercenaria mercenaria* expressing partial resistant to the eukaryotic QPX pathogen (91). Interestingly, tyrosine-protein phosphatase 10-like (Bg35029) is over-expressed in category 1, and receptor-type tyrosine-protein phosphatase U-like (Bg28838) is over-expressed in category 2. Notably, 45 different receptor-type PTPs have been found among our response categories and await further study.

The SUS response network emphasized the 40 dpe time point, when a snail with a full-blown Sm infection is coping with the stresses of repeated cercarial production and elimination of those cercariae across epithelial surfaces. Consequently, it makes sense that genes like bactericidal permeability-increasing protein and C-type mannose receptor 2 that would likely bolster snail defenses to extraneous pathogens are prominent parts of the SUS network. The conspicuous involvement of unknowns in the SUS network highlights the work yet to be done. Identification of the unknown features involved may well prove to have general significance in understanding the interactions among thousands of species of digenetic trematodes and their respective molluscan hosts.

For genes identified in four GWMS or six feature-specific studies of putative resistance factors (**Table S3**), we examined their representation among our seven response categories. In general, for both kinds of studies, about half of all genes were not responsive to Sm exposure. For the GWMS studies, the Sm responsive genes were about evenly split between RES and SUS snails, whereas feature-specific studies favored RES snails. Our category 4 revealed the most Sm responsive genes, and though it varied somewhat from study to study, comprised a significant proportion of the responsive snails for both GWMS and feature-specific studies. FREPs/FReDs were particularly numerous in category 4. The GWMS studies identified more Sm responsive genes in category 7 than category 6, potentially indicative of factors that when up-regulated late in infection prevent cercariae production such that the snails expressing these genes were scored as being “resistant” by the criterion used in the GWMS studies. This highlights that there is more than one way to identify resistance in Bg exposed to Sm: one refers to the early response to recently-penetrated miracidia resulting in encapsulation and destruction of the parasite with a few days post-exposure; another is that the development of the parasite proceeds but is slowed such that the production of sporocysts and cercariae is delayed or reduced. Our category 4 provides a view of the first kind of resistance and the evidence is good that the resistant BS-90 snails used display this type of response (38). Our category 7 provides a possible view of some of the contributing molecules that might contribute to the second kind of resistance, though all the SUS snails studied at 40 dpe in our study were shedding cercariae.

The results of this study provide a great deal of raw material for further investigation of the phenomenon of resistance. Given the multiplicity of genes seemingly involved, or potentially involved, including members of several gene families like FREPs/FReDs, AIGs/GIMAPs, C-type lectins, GPCRs, PTPs, biomphalysins and TEPs, and a host of largely unknown features awaiting further characterization, it is perhaps not surprising that strong consensus has not emerged among the results of various GWMS, or between feature-driven immunological approaches and GWMS. Among possible contributing factors are that the starting strains of Bg and parasites have differed among labs, that early vs. later-acting forms of resistance might be targeted for investigation, and that the resistance process itself likely unfolds in multiple stages, perhaps initially involving receipt of signals that activate multiple kinds of cell surface receptors that in turn activate signaling pathways resulting in production downstream of large quantities of complex suites of effectors. In cases where the snail may be harboring a considerable burden of Sm parasites, other confounding immune responses may also be initiated that help protect the schistosome-compromised snail from other pathogens, including many other trematode species as well as largely uncharacterized viruses and both prokaryotic and eukaryotic parasites capable of infecting snails. The transcriptomic data provided here can also provide new insights regarding other key aspects of the Bg-Sm interaction, perhaps most likely helping to identify genes and suggest mechanisms involved in the castration so commonly experienced by molluscs infected by trematodes.

## Conclusions

1) For both Bg strains, many features are listed as ncRNAs or as uncharacterized proteins, including many that are prominently expressed, indicating there is much to learn about the snail response to trematodes; 2) Constitutive differences in several putative immune-related genes were noted between RES and SUS snails, with RES showing overexpression of more than SUS; 3) The SUS response to Sm at 0.5, 2 and 8 dpe was relatively modest, suggestive of an ability of Sm to evade detection rather than of potent immune inhibition; 4) SUS snails at 40 dpe harbor massive cercariae-producing infections and revealed both many strongly down-regulated genes suggestive of Sm-mediated suppression or manipulation and several strongly up-regulated responses suggestive of protective or tolerance-like responses; 5) RES snails responded very strongly to Sm at both 0.5 and 2 dpe with up-regulation of many immune/resistance related genes. By 8 and especially 40 dpe, when parasites were gone, their responses were negligible; 6) Some genes identified by genome-wide mapping studies or immune feature-specific studies were constitutively different between SUS or RES, some were nonresponsive to Sm and some were responsive in ways indicative of early expression in RES or late tolerance in SUS snails; 7) Both network analysis and the expression of specific suites of lectins and G protein-coupled receptors in snails of categories 4, 6 and 7 revealed the responses to Sm of both SUS and RES snails to be orchestrated, and distinctly different; and 8) This study provides the most thorough transcriptomic comparison of RES and SUS snails to date, revealing many new candidate resistance genes as well as providing reinforcing support for the role of several previously identified genes in the response of Bg to Sm.

## Data Availability Statement

**﻿** The raw RNA sequencing data are available at NCBI under SRA accession: PRJNA591872. All other data generated or analyzed during this study are included in this published article and its **Supplementary Information** files.

## Author Contributions

**﻿**EL, LL, and S-MZ conceived and designed the experiments. LL conducted the RNA-Seq experimental work. LL and LB analyzed the data. EL, LL, LB, S-MZ and SB carried out the interpretation of the data. EL, LL, and LB drafted the manuscript and revised the draft paper. All authors read and approved the final manuscript.

## Funding

**﻿**This research was funded by NIH awards R37AI101438 (EL) and AI132953 (S-MZ). This research was also facilitated by the Center for Evolutionary and Theoretical Immunology (CETI) supported by NIH COBRE award 5P30GM110907–05. Funding agency played no role in the study design, data analysis and interpretation, or in writing the manuscript.

## Conflict of Interest

The authors declare that the research was conducted in the absence of any commercial or financial relationships that could be construed as a potential conflict of interest.

## Publisher’s Note

All claims expressed in this article are solely those of the authors and do not necessarily represent those of their affiliated organizations, or those of the publisher, the editors and the reviewers. Any product that may be evaluated in this article, or claim that may be made by its manufacturer, is not guaranteed or endorsed by the publisher.

## Supplementary Material

Supplementary Figure 1 Bar graph of 3 Differential Expression (DE) methods comparison.

Supplementary Figure 2 Plot of 45 modules in RES strain for co-expression network analysis.

Supplementary Figure 3 Plot of 29 modules in SUS strain for co-expression network analysis.
