# Supplementary material for: An Overview of Transcriptional Responses of Schistosome-Susceptible (M line) or -Resistant (BS-90) Biomphalaria glabrata Exposed or Not to Schistosoma mansoni Infection
Source: Front Immunol. 2022 Jan 12;12:805882. doi: 10.3389/fimmu.2021.805882 (PMC8791074; doi:10.3389/fimmu.2021.805882)
Supplement: Supplementary file 4 [file Table_1.docx]

**Table S1.** **Overview of studies on comparisons of parasite-susceptible and -resistant strains of *Biomphalaria glabrata*: constitutive differences and differing responses to various kinds of immune stimuli**

| **Reference** | **Susceptible Strain Used** | **Resistant Strain Used** | **Parasite Used for Strain Selection** | **Immune or Stress Stimuli Employed** | **Brief Summary of Strong/Noteworthy Responding Genes** |
| --- | --- | --- | --- | --- | --- |
| Adema et al., 1997 (1) | M line strain | 13-16-R1, two Brazilian isolates (Salvador Bahia, Belo Horizonte) strains | *S. mansoni* | *E. paraensei* | Refer to their Figures 2&3, a FREP (fibrinogen-related protein) family in *B. glabrata* was reported; lectins and involved in the innate defense. |
| Adema et al., 2010 (2) | M line strain |  | *S. mansoni, E. paraensei* | Wounding, injection of *E. coli*, *M. luteus*, *S. mansoni*, or *E. paraensei* | Refer to their Table 3, list of genes differentially expressed after exposure to different immune challenges, including FREP2, 4, 6, 7, 8, SOD1, Serpin B4, Serpin B6, multi-drug resistance protein 2, NF-kappa-B p105 subunit, C1q-like protein 4, BgLBP/BPI, MAP kinase 2, etc. |
| Adema et al., 2017 (3) | BB02 strain |  | *S. mansoni* |  | Refer to their supplementary materials, the authors characterized the genome of *B. glabrata* BB02 strain and described aspects of phero-perception, stress responses, immune function and regulation of gene expression that support the persistence of *B. glabrata* in the field. |
| Allan et al., 2017 (4) | BgGUA SS strain (collected from Guadeloupe) | BgGUA RR strain (collected from Guade-loupe) | *S. mansoni* | SmGUA (collected from Guadeloupe) | Refer to their Figures 3&4, the expression of *grctm6*, a gene encoding the Guadeloupe Resistance Complex (GRC) Transmembrane 6 (Grctm6) protein was analyzed in-depth. The constitutive *grctm6*mRNA levels in resistant whole snail, head-food, albumen gland and hemolymph were investigated. |
| Allan et al., 2019 (5) | Outbred BgGUA SS strain | Outbred BgGUA RR strain | *S. mansoni* |  | Refer to their Table 1, the levels of 13 identified hemolymph proteins differ significantly between homozygous RR and SS GRC genotypes of BgGUA. They contain tubulin beta chain, tubulin alpha chain, actin (cytoplasmic), histone H4, schistosomin, BgMFREP5, fibrinogen-related protein J3, granulin-like protein, heat shock protein 70, fructose-bisphosphate aldolase, elongation factor 1-alpha-like isoform X1, malate dehydrogenase, ubiquitin. |
| Allan et al., 2020 (6) | Guadeloupe strain (BgGUA) |  | *S. mansoni* | Temperature (20C, 26C, or 32C), phorbol 12-myristate 13-acetate (PMA) or DPI (PMA + Diphenyleneiodonium) | Refer to their Fig 2, hydrogen peroxide production by BgGUA hemocytes (mRNA levels) was elevated in some treatments. |
| Baron et al., 2013 (7) | Albino strain |  | *S. mansoni* | *S. mansoni,*  *M. luteus,*  *P. aeruginosa, B. cereus,*  *C. freundii,*  *C. albicans,*  *S. cerevisiae,*  *E. coli SBS363* | Refer to their Figure 1, the relative expression levels of *B. glabrata* BgLBP/BPI1 (lipopolysaccharide binding protein/bactericidal permeability increasing protein) transcripts in the albumen gland (AG), hepatopancreas (Hp), head-foot (HF), gonads (G), digestive tract (DT) and circulating hemocytes (HC) were determined by using real-time quantitative PCR. The expression studies showed that the major sites of expression for BgLBP/BPI was the albumen gland. |
| Baron et al., 2016 (8) | Albino strain |  | *S. mansoni* | *E. coli*, *Bacillus cereus*, and *Saccharomyces cerevisiae* | Refer to their Table 3, four additional LBP/BPI from *B. glabrata* were characterized, which highlights the importance of LBP/BPI in invertebrate immunity. |
| Bender et al., 2007 (9) |  | 13-16-R1 strain | *S. mansoni* | *S. mansoni* | Refer to their Figure 1, individual 13-16-R1 snails possessing the allele previously associated with resistance to *S. mansoni* expressed significantly higher levels of hemocyte *SOD1* transcripts than individuals lacking the allele. |
| Bouchut et al., 2006 (10) | EAF strain | CB strain | *E. caproni* | *E. caproni* | Refer to their Figure 1, 4 genes whose transcripts were differentially represented between hemocytes from resistant CB strain and susceptible EAF strain. These genes encode two dermatopontin-like, one matrilin-like and one cadherin-like proteins. Expression of exposure to *E. caproni* showed that dermatopontins may be involved in the compatibility differences between these strains. |
| Bouchut et al., 2006 (11) | EAF strain | CB strain | *E. caproni* | *E. caproni* | Refer to Figures 7&8, expression of five genes encoding an aldolase, an intermediate filament protein, a cytidine deaminase, the ribosomal protein P1 and the histone H4 respectively was investigated in parasite-exposed or unexposed snails. |
| Bouchut et al., 2007 (12) | EAF strain | CB strain | *E. caproni* | *E. caproni* | Refer to their Table 3&4, Figure 3&4, some candidate transcripts (calcium-binding proteins and glycolytic enzymes) and novel candidate transcripts were differentially expressed between strains of *B. glabrata*. Some newly identified genes were revealed involving in immune processes encoding proteases, protease inhibitors, a lectin, and aplysianin-like molecule, and cell adhesion molecules. |
| Bridger et al., 2018 (13) | NMRI,  BB02 strain | BS-90 strain | *S. mansoni* | *S. mansoni* | Refer to their Figure 2, piwi RNA was differentially regulated in *S. mansoni* infected in *B. glabrata* snails depending on their susceptibility phenotypes. |
| Cantinha et al., 2017 (14) |  | *B. glabrata* (Belo Horizonte strain) | *S. mansoni* | Cadmium | Refer to their Figure 2&3, induction of the protein HSP70 expression relative to the control group values in *B. glabrata* snails exposed to CdCl_2_. |
| de Melo et al. 2020 (15) | Brazilian strain (from Pernambuco, Brazil) |  | *S. mansoni* | *S. mansoni* | Refer to their Fig 4, FREP3 and FREP4 were differentially expressed after exposure to *S. mansoni*. |
| Deleury et al., 2012 (16) | EAF strain | CB strain | *E. caproni* | *E. coli*,  *B. cereus*,  *S. cerevisiae* | Refer to their Figure 7 and supporting Figure S2, some protein families such as GNBP, TEPs, calmodulins, ferritins, or cyclophilins are expressed after any immune challenges. |
| Dheilly et al., 2015 (17) | BgBRE strain |  | *S. mansoni* |  | Refer to their Figure 1&2, the RNA-Seq data reveled some new FREP subfamilies and FREP12 subfamily, C-type lectin-related proteins (CREPs), and Galectin-related protein (GREP) at constitutive level. |
| Dinguirard et al. 2018 (18) | NMRI strain | BS-90 strain | *S. mansoni* | *S. mansoni* | Refer to their Tables 1-4, big differences in proteins expressed by susceptible NMRI and resistant BS-90 snail hemocytes to *S. mansoni* sporocysts during active encapsulation were detected. NMRI hemocytes were exhibiting extensive downregulation of protein expression and a lower level of constitutively expressed immune-relevant proteins compared to BS-90. |
| Fogarty et al., 2019 (19) | NMR1 strain |  | *S. mansoni* | *S. mansoni* | Refer to their Tables 1&2, high confidence non-redundant ESPs (excretory-secretory proteins) with putative anti-pathogen function identified in resistant *B. glabrata* snail-conditioned water (SCW). For instance: Leukocyte elastase inhibitor-like, haemoglobin type-1, superoxide dismutase [Cu-Zn], Adenosine deaminase CECR1, Glutathione peroxidase-like, heat-shock 70 kDa protein cognate 4, enolase-phosphatase E1, Peroxiredoxin1, Rho GDP-dissociation inhibitor 1, etc. |
| Galinier et al., 2013 (20) | Brazil strain |  | *S. mansoni* | *S. mansoni* from Brazil | Refer to their Figure 10, the expression of biomphalysin, a ß pore-forming toxin (ß-PFT) is restricted to immune cells (*B. glabrata* embryonic cells, Bge) and is not induced by *S. mansoni* challenge. |
| Galinier et al., 2017 (21) | BgVEN,BgGUA, BgBRE, BgBAR strain |  | *S. mansoni* | *S. mansoni* | Refer to their Figures 6&7, the expression levels of FREPs and SmPoMucs could be related to the compatibility level of *S. mansoni*. |
| Goodall et al., 2004 (22) | M line Oregon strain | 13-16-R1 strain | *S. mansoni* | *S. mansoni* | Refer to their Figures 2 and 3, different constitutive levels of Cu/Zn SOD in the 13-16-R1 and M line strains were identified. |
| Granath et al., 1994 (23) | M line strain | 10-R2, 13-16-R1 strain | *S. mansoni* | *S. mansoni* | Refer to their Figure 2 and Discussion, plasma IL-1 (interleukin 1) levels were significantly affected by exposure to *S. mansoni*, with levels dropping in M line and 10-R2 snails but increasing in the 13-16-R1 strain. Both resistant strains of 10-R2 and 13-16-R1 maintained significantly higher IL-1 levels than susceptible M line snails. |
| Guillou et al., 2004 (24) | EAF strain | CB strain | *E. caproni* | *E. caproni* | Refer to their Figure 4 and Discussion, BgSel (a candidate lectin) expression level was high in EAF snails and almost undetectable in CB strain, and exposure to *E. caproni* did not affect the expression level of BgSel in either strain. |
| Guillou et al., 2007 (25) |  | CB strain | *E. caproni* | *E. caproni* | Refer to their Table 2, 10 candidates of interest were identified, including GST, SOD, LBI/BPI, cystatins, calcium-binding proteins, and C-type lectins. |
| Hanelt et al., 2008 (26) | M line strain |  | *S. mansoni* | PR-1 *S. mansoni,*  *E. coli* or *M. luteus* | Refer to their Tables 1&2, a number of ORESTES were differentially expressed in *B. glabrata*. Functions of the ORESTES were related to pattern recognition/binding, cell adhesion, signal transduction, antioxidant related, apoptosis related, stress response, metal-binding serpins, proteases, inflammatory response, and putative immune factors. |
| Hanington et al., 2010 (27) | M line strain |  | *S. mansoni* | *S. mansoni*  *E. paraensei* | Refer to their Table 2, FREP4 was significantly elevated throughout the course of infection with either parasite, effectively serving as a marker of infection. Several fibrinogen-containing lectins and homologs of molecules best known from vertebrate phagocytic cells were persistently down-regulated. |
| Hanington et al., 2012 (28) | M line strain | BS-90 strain | *S. mansoni* | *S. mansoni*  *E. paraensei* | Refer to their Figure 5, several transcripts (FREP2, FREP3, FREP4, coagulation factor X1, dermatopontin, dual oxidase, galectin-4, HSP70, MIF, Martrilin-1 A, peroxiredoxin, Serpin B4, SOD Cu-Zn A, SOD Cu-Zn B) were increased or decreased in expression in BS-90 immunocompromised by irradiated *E. paraensei* before challenge with *S. mansoni*. |
| Hathaway et al., 2011 (29) | BB02 strain, M line strain |  | *S. mansoni* |  | Refer to their Table 1, Figure 2, 16 out of 20 major *B. glabrata* egg mass fluid (EMF) polypeptides were identified as defense-related, including protease inhibitors, a hemocyanin-like factor and tyrosinase, extracellular Cu-Zn SOD, C-type lectins, Gram-negative bacteria-binding protein (GNBP), aplysianin/achacin-like protein, lipopolysaccharide binding protein/bacterial permeability-increasing proteins (LBP/BPI) that differed from those previously described from hemocytes. |
| Hertel et al., 2005 (30) | M line strain | BS-90 strain | *S. mansoni*  *E. paraensei* | *S. mansoni*  *E. paraensei* | Refer to their Figure 1, FREP2 and 4 were significantly elevated in M line and BS-90 after exposure to *E. paraensei*. FREP2 and 4 were up-expressed in BS-90 after *S. mansoni* exposure. |
| Ittiprasert et al., 2009 (31) | NMRI strain, LAC2 line | BS-90 strain | *S. mansoni* | Heat shock, irradiated or normal *S. mansoni* miracidia | Refer to their Figures 2, 4&5, stress-related genes of heat shock protein (Hsp70), reverse transcriptase (RT) were significantly co-induced early in NMRI snails, but not in BS-90 and LAC2 strains. These transcripts were down-regulated upon exposure to *S. mansoni* although penetration behavior or irradiated vs. normal *S. mansoni* were the same, indicating that regulation of Hsp70 and RT was elicited by infection and not injury. |
| Ittiprasert et al., 2010 (32) | NMRI strain, LAC2 line | BS-90 strain | *S. mansoni* | Normal or attenuated *S. mansoni* miracidia | Refer to their Table 2, Figures 2&3, Several transcripts were highlighted in the expression study. Fibrinolytic C terminal domain, cytidine deaminase, macrophage expressed gene 1, theromacin and Fas remained up-regulated in snails no matter if exposed to normal or attenuated *S. mansoni*. And C-type lectin and low-density lipoprotein receptor were induced only by exposure to normal miracidia. |
| Jiang et al., 2006 (33) | M line strain,  BS-90 strain | BS-90 strain | *E. paraensei*,  *S. mansoni* | *E. paraensei*,  *S. mansoni* | Refer to their Figures 1-8, in vivo and in vitro knockdown of FREP2 gene expressions in *B. glabrata* by using RNAi were documented. Expression of house-keeping gene myoglobin in BS-90 was significantly reduced after introduction of myoglobin dsDNA relative to non-RNAi treated snails. |
| Jones et al., 2001 (34) |  | *B. glabrata* strain 1778 (Belo Horizonte, Brazil) | *S. mansoni* | *S. mansoni* | Refer to their Figure 3, the transcript of HSP-70 in resistant strain of *B. glabrata* was confirmed to be upregulated in the exposure to *S. mansoni* by using semi-quantitative RT-PCR with primers specific to the sequenced HSP70-like fragment. |
| Jung et al., 2005 (35) | M line, BS-90 strain | BS-90 strain | *E. paraensei*,  *S. mansoni* | *E. paraensei,*  *S. mansoni* | Refer to their Figure 1, susceptible snails increased expression of MnSOD following infection with *E. paraensei* or *S. mansoni*, and expression was reduced in the incompatible combination (BS-90 *B. glabrata* and *S. mansoni*). Concluded expression of MnSOD is consistent with its involvement in a stress response of *B. glabrata*. |
| Kaur et al., 2016 (36) | BB02 strain |  | *S. mansoni* | Chemicals: 5α-dihydro-testosterone (DHT) or 17 α-methyl-testosterone (MT) | Refer to their Figure 4, normalized gene expression of individual albumen glands from 125ng/l-MT and solvent-control groups was investigated. The normalized gene expression in individual snails for 5 genes was identified as differentially expressed in the albumen glands of exposed and control groups. Also, no significant differences between tissues from control and exposed snails were identified. |
| Knight et al., 2009 (37) | NMRI, M line, LAC2 strain, | BS-90 strain | *S. mansoni* | *S. mansoni* | Refer to Figure 4, the quantitative real-time RT-PCR showed that in the expression level of *BgPrx4* (*B. glabrata* 2-Cys peroxiredoxin gene) transcript induction following infection, with the transcript up-regulated in BS-90 snails during the early phase (5 hours) of infection compared to M line and NMRI snails in which it was down-regulated within the early time points. |
| Knight et al., 2011 (38) | NMRI strain |  | *S. mansoni* |  | Refer to their Figures 3-5, relative expression of Cathepsin B (CathB), Myo and peroxiredoxin (Prx) of NMRI snail with small interfering (si) RNA or knockdown technique versus untreated snails was investigated. |
| Knight et al., 2015 (39) | NMRI strain | BS-90 strain | *S. mansoni* | *S. mansoni* (NMRI) strain, heat stress | Refer to their Figure 5, expression levels of the heat shock protein (Hsp) 70 showed that the protein is differentially expressed between NMRI and BS-90 strains, with NMRI snails expressing more protein than BS-90 after early exposure to wild-type *S. mansoni* but not to radiation-attenuated *S. mansoni* miracidia. |
| Knight et al., 2016 (40) | NMRI strain | BS-90 strain | *S. mansoni* | attenuated or normal *S. mansoni* (NMRI) strain | Refer to their Figure 3, the expression of the *B. glabrata* HSP70 gene in the interphase nuclei of cells derived from NMRI and BS-90 snail strains, pre- and post-exposure to *S. mansoni* miracidia. *B. glabrata* were infected with various ways: wild type *S. mansoni*, irradiated attenuated *S. mansoni* miracidia, dissected, fixed or subjected fluorescent in situ hybridization (FISH). No significant change in expression was observed in BS-90 strain snails, and no real HSP70 expression was detected by qPCR when NMRI and BS-90 strains were infected with irradiated miracidia. |
| Larson et al., 2014 (41) |  | 13-16-R1 strain | *S. mansoni* | *S. mansoni* | Refer to their Figure 3, allograft inflammatory factor (aif), the gene for the inferred phox subunit of nicotinamide adenine dinucleotide phosphate (NADPH) oxidase (infPhox) and peroxiredoxin 1 (prx1) were expressed at higher levels in resistant snails. |
| Lassalle et al. 2020 (42) | Albino Brazilian strain (BgBRE) |  | *S. mansoni* | Gram-positive, Gram-negative bacteria and yeast, *S. mansoni* | Refer to their Fig 7, Glabralysins expression in response to different pathogens or unexposed were monitored at six time points (3, 6, 12,24, 48, and 96hrs) with *Micrococcus luteus*, *Saccharomyces cerevisiae, Escherichia coli,* and *S. mansoni.* |
| Lehr et al., 2010 (43) | *B. glabrata* strain Puerto Rico (*Bg*PR) | Salvador (*Bg*BS-90) | *S. mansoni* | Puerto Rican isolate of *S. mansoni* | Refer to their Figures 5&7, an enhanced expression of serologically cross-reacting, fucosylated carbohydrate epitopes by the highly susceptible *Bg*PR strain in comparisons to the resistant *Bg*BS-90 snail was documented. The immunohistochemical studies also showed that an enhanced expression of such carbohydrate epitopes in susceptible *Bg*PR snail tissue. The study provide evidence for a correlation of *B. glabrata* susceptibility towards *S. mansoni* infection and the expression of carbohydrate determinants shared by *S. mansoni* and *B. glabrata* snails. |
| Li et al., 2020 (44) | M line strain | BS-90 strain | *S. mansoni* | *S. mansoni* | ﻿Refer to their results, the association between humoral immune molecules in *B. glabrata*, including: BgFREP3, BgTEP1, BgFREP2 and Biomphalysin were addressed. |
| Li et al., 2021 (45) | M line strain | BS-90 strain | *S. mansoni* |  | Refer to their results, transcripts of single-cell RNA-seq from *B. glabrata* M line or BS-90 single granulocytes and hyalinocytes, from snails not exposed to *S. mansoni* nor given other immune stimulation were examined. This transcriptomic study highlighted the role of hyalinocytes as producers of immune effectors including biomphalysin and TEPs. It’s also confirmed that the granulocytes and hyalinocytes of BS-90 snails are generally more “immunologically prepared” than *S. mansoni*-susceptible M line *B. glabrata*. |
| Lockyer et al., 2000 (46) |  | *B. glabrata* strain 1778 (Belo Horizonte, Brazil) | *S. mansoni* | *S. mansoni* (Kenyan strain) | Refer to their Results and Discussion, a few transcripts were identified, down regulation of a 241 bp mRNA expressed sequence fragment - with an open frame showing 48% identity to a *B. glabrata* cytochrome p450 over 80 residues, which has been confirmed by using semi-quantitative RT-PCR. |
| Lockyer et al., 2004 (47) | NHM1742 strain | NHM1981 strain | *S. mansoni* | *S. mansoni* (Belo Horizonte Strain) | Refer to their Tables 2, 5 transcripts including HSP70 and several novel transcripts with one containing at least two globin-like domains were differentially expressed in *B. glabrata* by semi-quantitative RT-PCR. |
| Lockyer et al., 2007 (48) | NHM1742 strain | NHM 3017, derived from BS-90 | *S. mansoni* | *S. mansoni* (Belo Horizonte strain) | Refer to their Figure 4 and Discussion, two stress response genes: ferritin and serine protease HtrA2 would be expected to be simulated in response to schistosome challenge, and up-regulated in resistant snails. |
| Lockyer et al., 2007 (49) | NHM1742, BB02 strain | BS-90 strain | *S. mansoni* | *S. mansoni* (Belo Horizonte strain) | Refer to their Table 3, a long list of differentially expressed transcripts was selected by gene ontology. Individual *B. glabrata* ESTs were identified antioxidant proteins, signal transducers, transcriptional regulators and immune or stress responses proteins. |
| Lockyer et al., 2008 (50) | NHM1742 strain | NHM3017, derived from BS-90 strain | *S. mansoni* | *S. mansoni* | Refer to their Tables 2&3, 98 differentially expressed genes or gene clusters were identified, 94 resistant-associated and 4 susceptible-associated. Resistant strain-specific expression genes include cathepsin L, ornithine decarboxylase, HSP70, importin 7, copine 1, cytoplasmic intermediate filament (IF), elongation factor 1α and EF-2. |
| Lockyer et al., 2012 (51) | NHM1742 strain | NHM3017 strain | *S. mansoni* | *S. mansoni* | Refer to their Figure 4 and supporting file S2, genes were identified as significantly differentially expressed between resistant and susceptible *B. glabrata*, before exposure, after exposure to *S. mansoni* and both before and after. |
| Lu et al., 2020 (52) | M line strain | BS-90 strain | *S. mansoni* | *S. mansoni* | Refer to their Fig 5 &Table 1, *B. glabrata* AIG (avrRpt2-induced gene) genes were differentially expressed between SUS and RES strains comparison at constitutive level, and responses to *S. mansoni* following exposure to infections. |
| Lu et al., 2020 (53) | M line strain | BS-90 strain | *S. mansoni* | *S. mansoni* | Refer to their Table 3, *B. glabrata* FReD genes (containing a fibrinogen domain) were differentially expressed between SUS and RES strains, comparison at constitutive level, and responses to *S. mansoni* following exposure to infections. |
| Mendes et al., 2019 (54) | Belo Horizonte strain |  | *S. mansoni* | *S. mansoni* | Refer to their Tables 1,2 & Fig 4, lists of proteins were differentially expressed in *B. glabrata* 24 hrs after exposure to *S. mansoni*. |
| Mendes et al., 2020 (55) | Brazilian strain |  | [*Angiostrongylus cantonensis*](https://www.sciencedirect.com/topics/agricultural-and-biological-sciences/angiostrongylus-cantonensis) | [*Angiostrongylus cantonensis*](https://www.sciencedirect.com/topics/agricultural-and-biological-sciences/angiostrongylus-cantonensis) | Refer to their Table 1,2, a few proteins were differentially expressed in in *B. glabrata* 24 hrs after exposure to *A. cantonensis*. |
| Mitta et al., 2005 (56) | EAF strain | CB strain | *E. caproni* | *E. caproni* | Refer to their Table 2 and Figure 2, cystatin, dermatopontin/LAF, matrilin, AIF, LBP and serine protease inhibitor-like molecules were regularly and highly expressed following infection in resistant snails. Dual oxidase, FREP3, β-integrin, F-spondin, α-trypsin inhibitors and integrin α-5-like showed constitutive differences in the expression levels between the two strains. |
| Moné et al., 2011 (57) | BgBRE, BgGUA strain |  | *S. mansoni* | *S. mansoni* (a Brazilian strain *Sm*BRE, a Guadeloupean strain *Sm*GH2) | Refer to their Figures 4-6, expression of intracellular reactive oxygen species (ROS), Hydrogen peroxide (H_2_O_2_) in *B. glabrata* hemocytes were measured. The constitutive level of superoxide anion plasma content in *B. glabrata* Guadeloupean strain, *Bg*GUA, versus Brazilian strain, *Bg*BRE was compared. |
| Myers et al., 2008 (58) | M line, NMRI strain | BS-90 strain | *S. mansoni* | *S. mansoni* (NMRI) strain | Refer to their Figure 4, expression of the snail hepatopancreas cathepsin B transcript in resistant (BS-90) and susceptible (NMRI and M line) pre- and post-exposure to *S. mansoni* was investigated. Higher up-regulation of cathepsin B transcript in BS-90 than in M line and NMRI snail after parasite exposure was observed. |
| Nelson et al., 2016 (59) | M line strain | Salvador strain | *S. mansoni* | *S. mansoni,* abnormal temperature, starvation stress | Refer to their Figure 6, exposure to 33C for 4~5 hrs triggered immediate upregulation of heat shock protein (HSP) 70 and 90 expression in resistant strain (Salvador), but did not alter resistance, and Heat shock protein expression levels returned to baseline after 2 weeks at 33C. |
| Nowak et al., 2004 (60) | M line strain | BS-90 strain | *S. mansoni* | *S. mansoni* | Refer to their Table 1, cytochrome C oxidase subunit VIb, Fibrinogen related protein 2, stefin/cystatin, thrombospondin related protein, serine protease inhibitor 6, ATP synthase α subunit were strong candidate for differential expression in the BS90 strain. |
| Pila et al., 2016 (61) | M line strain | BS-90 strain | *S. mansoni* | *S. mansoni* | Refer to their Figure 2A&B, different expression of BgGRN (*B. glabrata* granulin) was observed after 1h in BS-90 snails. BgGRN also increased in abundance following challenge of *B. glabrata* M line with *S. mansoni*. |
| Pila et al., 2016 (62) | M line strain | BS-90 strain | *S. mansoni* | *S. mansoni* (NMRI) strain | Refer to their Figure 1, the resistant strain BS-90 displayed up-regulation of *B. glabrata* Toll-like receptor (BgTLR) relative to M line. Transcript expression of BgTLR was discovered to be very responsive in BS-90 snails after exposure to *S. mansoni*, but expression in M line snails was not significantly increased. |
| Pinaud et al., 2016 (63) | BgBRE strain |  | *S. mansoni* | SmBRE | Refer to their Figures 3&5, lists of immune-related candidates and plasma factors; innate immune memory responses were discovered by RNA-Seq. |
| Pinaud et al., 2019 (64) | BgBRE strain |  | *S. mansoni* | *S. mansoni* (SmBRE, SmVEN)*,*  *S. rodhaini* (SROD) | Refer to their Figures 2&3, immune recognition-related transcripts following homologous or heterologous challenge were listed, including selectin, C-type lectin, tenascin, FREP3.3, galectin, TEP, C1q-like proteins, etc. |
| Pinaud et al., 2021 (65) | Albino Brazilian strain (BgBRE) |  | *S. mansoni* | *S. mansoni*, Gram-positive, Gram-negative bacteria or yeast | Refer to their Fig 8 & Table 2, a few biomphalysin genes were detected differentially expressed after exposure to *S. mansoni*, bacteria or yeast. |
| Portet et al. 2018 (66) | Albino Brazilian strain (BgBRE) |  | *S. mansoni* | Gram-positive, Gram-negative bacteria and yeast, *S. mansoni* | Refer to their Fig 5, ﻿BgTEP expression was monitored at three time points (6, 12, and 24 h) post-challenge with *Micrococcus luteus, Escherichia coli, Saccharomyces cerevisiae,* and *Schistosoma mansoni*. |
| Portet et al., 2019 (67) | Albino Brazilian strain (BgBRE), Venezuelan strain (BgVEN) |  | *S. mansoni* | *S. mansoni*  *S. rodhaini* | Refer to their Figure 1 and Supporting Table S2 and S3, 6 parasite gene products have been identified involved in parasite modulation or suppression of snail immunity. These snail molecules correspond to heat shock proteins, GST, NADH dehydrogenase subunit, and calreticulin; alpha-2-macroglobulin, von Willebrand factor type EGF with pentraxin domain. |
| Portet et al., 2021 (68) | Albino Brazilian strain (BgBRE) |  | *S. mansoni* | *S. mansoni* | Refer to their Figures 6&S3, *B. glabrata* biomphamacins, LBP/BPI and achacins were detected differentially expressed at sampling time points after *S. mansoni* exposure. |
| Queiroz et al., 2017 (69) |  | *B. glabrata* (Belo Horizonte strain) | *S. mansoni* | *S. mansoni* | Refer to their Figures 6&7, Relative gene expression for 4 development times (5, 10, 20 and 40 days) in *B. glabrata* using egg masses as the baseline; five genes (Argonaute, Piwi, Drosha, Exportin 5, Tudor) were analyzed. The relative gene expression of *B. glabrata* at several time points of infection by *S. mansoni* using uninfected snails at the same time points as a baseline was documented. |
| Raghavan et al., 2003 (70) | M line strain | BS-90 strain | *S. mansoni* | *S. mansoni* (NMRI strain) | Refer to their Figures 1&5, the expression status of reverse transcriptase (RT) was investigated in BS-90 and M line respectively at pre- and post-exposure to *S. mansoni* miracidia by using protein extracts from the head-foot and posterior region tissues. It showed that RT activity was greater in posterior region than in the head-foot. After exposure, RT activity increased greatly in the head-foot, with the highest activity at 1-day post exposure. |
| Smith et al., 2021 (71) | NMRI strain | BS-90 strain | *S. mansoni* | *S. mansoni* (NMRI) strain, heat stress | Refer to their Figures 1-5, transcripts encoding proteins with a role in epigenetics, such as PIWI (*BgPiwi*), chromobox protein homolog 1 (*BgCBx1*), histone acetyltransferase (*BgHAT*), histone ﻿deacetylase (*BgHDAC*) and metallotransferase (*BgMT*) were highly expressed in those cultured at 25˚C. The expression of the non-LTR- retrotransposon (*nimbusRT*) encoding transcript at 120 min after infection of resistant BS90 piwi-siRNA treated snails was observed to be upregulated (Figure 11). |
| Tennessen et al., 2020 (72) |  | 13-16-R1 strain | *S. mansoni* | *S. mansoni* | Refer to their Figures and Supplementary File 1, genomic region PTC2 in *B. glabrata* exhibits the largest known correlation with susceptibility to *S. mansoni* infection. In the PTC2 region, 11 genes were identified, and most of these genes encode single-pass transmembrane (TM) proteins. |
| Tunholi-Alves et al., 2011 (73) | *B. glabrata* (Sumidouro, RJ isolate) |  | *E. paraensei* | *E. paraensei* (with different dose) | Refer to their Figure 1, the levels of expression of neutral lipids (cholesterol, cholesterol ester, triacylglycerol and fatty acids) in the digestive gland-gonad (DGG) complex of *B. glabrata* infected by 5 and 50 miracidia of *E. paraensei* relative to the control group were investigated. |
| Vergote et al., 2005 (74) | EAF-S strain | CB-R strain | *E. caproni* | *E. caproni* | Refer to their Figures 5-7, genes encoding two isoforms of a glycolytic enzyme, two isoforms of a calcium binding protein and an inhibitor of cysteine protease were characterized, and differential expression identified between two strains. |
| Zahoor et al., 2010 (75) | NHM1742 strain | NHM 3017, derived from BS-90 strain | *S. mansoni* | *S. mansoni* (Belo Horizonte strain) | Refer to their Figures 1, 2, 6, 8&9. Hemocytes from two *B. glabrata* strains showed reduced HSP70 protein levels following 1 hour challenge with *S. mansoni* excretory-secretory products (ESPs) when compared with unchallenged controls. |
| Zahoor et al., 2014 (76) | NHM1742 strain | NHM 3017, derived from BS-90 strain | *S. mansoni* | *S. mansoni* | Refer to their Figures 1&3, nuclear factor kappa B subunit Relish, elongation factor 1α, 40S ribosomal protein S9, and matrilin were shown as differentially expressed in resistant strain; cathepsins D and L, and theromacin were known as susceptible-snail specific genes. |
| Zhang et al., 2003 (77) | M line strain |  | *E. paraensei* | *E. paraensei* | Refer to their Tables 2&3, three additional FREP members FREPs 3.2, 12.1 or 13.1 were identified. |
| Zhang et al., 2007 (78) | M line strain | BS-90 strain | *E. paraensei*,  *S. mansoni* | *E. paraensei*,  *S. mansoni*,  *S. aureus*,  *E. coli*,  *S. cerevisiae* | Refer to their Figures 6&7, expression of *B. glabrata* peptidoglycan recognition proteins (BgPGRP) and *B. glabrata* gram-negative bacteria binding proteins (BgGNBP) was down-regulated in *B. glabrata* at 6 hour after exposure to three types of bacteria. No significant changes in expression were observed in snails at 2 days post-exposure to *E. paraensei* or *S. mansoni*. Up-regulation of BgPGRP-SA in M line snails at later time points of infection with *E. paraensei* (12- and 17-days post-exposure) was detected. |
| Zhang et al., 2008 (79) | M line strain | BS-90 strain | *S. mansoni* | *S. mansoni*  *E. paraensei* | Refer to their Figures 4, 6&7, two newly identified FBG-bearing protein encoded genes (*FReM* and *FREP14*) were found and comparative expression profiling of the two new genes and five FREP members (*FREP2, 3, 4, 12,* and *13*) during ontogenesis and a later stage of trematode infection was undertaken |
| Zhang et al., 2008 (80) | M line strain | BS-90 strain | *E. paraensei*,  *S. mansoni* | *E. paraensei*,  *S. mansoni*,  *S. aureus*,  *E. coli*,  *S. cerevisiae* | Refer to their Figures 3-6, the expression of multiple FREPs including FREP4 in plasma from M line and BS-90 snails is significantly up-regulated after infection with *E. paraensei*. The study also demonstrated that FREPs can bind *E. paraensei* sporocysts and their secretory/excretory products, and a variety of microbes (G+ and G- bacteria, and yeast) |
| Zhang et al., 2009 (81) | M line strain | BS-90 strain | *E. paraensei*,  *S. mansoni* | *E. paraensei*,  *S. mansoni*,  *S. aureus*,  *E. coli*,  *S. cerevisiae* | Refer to their Figures 4&5, expression of schistosomin was in higher abundance in embryos and juveniles relative to mature snails. Also, infection with *S. mansoni* and *E. paraensei*, did not provoke elevated expression of schistosomin in *B. glabrata* from early-stage infection to patent infection. |
| Zhang et al., 2011 (82) | M line strain | BS-90 strain | *S. mansoni* | *S. mansoni* | Refer to their Figures 9&10, expression of five transcription factors (TFs) in whole body tissue or pooled hemocytes of M line and BS-90 snails after exposure to *S. mansoni* was investigated. Five homologues of TFs from *B. glabrata*, designated BgRLISH, BgRel, BgSTAT1, BgSTAT2 and BgCREB, were identified and characterized in this study. |
| Zhang et al., 2015 (83) | M line strain |  | *S. mansoni* | Niclosamide (molluscicide) | Refer to their Table 1 and Figs 3&4, genes encoding cytochrome P450s (CYP), GST, multi-drug resistance protein, solute linked carrier, heat shock protein (HSP20, HSP40 and HSP70), ADP-ribosylation factor, cAMP response element-binding protein (CREB) and coatomer were upregulated in snails followed exposure to Niclosamide. |
| Zhang et al., 2016 (84) |  | Salvador strain | *S. mansoni* | Lipopolysaccharide (LPS), peptidoglycan (PGN), fucoidan (FCN) | Refer to their Tables 1&2, lists of differentially expressed genes elicited by LPS, PGN and FCN. Significant up-regulation was observed in four GiMAPs (GTPase of immunity-associated protein) genes and arginase. |

**References:**

1. Adema CM, Hertel LA, Miller RD, Loker ES. A family of fibrinogen-related proteins that precipitates parasite-derived molecules is produced by an invertebrate after infection. *Proc Natl Acad Sci* (1997) **94**:8691–6.

2. Adema CM, Hanington PC, Lun CM, Rosenberg GH, Aragon AD, Stout BA, Lennard Richard ML, Gross PS, Loker ES. Differential transcriptomic responses of *Biomphalaria glabrata* (Gastropoda, Mollusca) to bacteria and metazoan parasites, *Schistosoma mansoni* and *Echinostoma paraensei* (Digenea, Platyhelminthes). *Mol Immunol* (2010) **47**:849–860. doi:10.1016/j.molimm.2009.10.019

3. Adema CM, Hillier LW, Jones CS, Loker ES, Knight M, Minx P, Oliveira G, Raghavan N, Shedlock A, do Amaral LR, et al. Whole genome analysis of a schistosomiasis-transmitting freshwater snail. *Nat Commun* (2017) **8**:15451. doi:10.1038/ncomms15451

4. Allan ERO, Tennessen JA, Bollmann SR, Hanington PC, Bayne CJ, Blouin MS. Schistosome infectivity in the snail, *Biomphalaria glabrata*, is partially dependent on the expression of Grctm6, a Guadeloupe Resistance Complex protein. *PLoS Negl Trop Dis* (2017) **11**:1–15. doi:10.1371/journal.pntd.0005362

5. Allan ERO, Yang L, Tennessen JA, Blouin MS. Allelic variation in a single genomic region alters the hemolymph proteome in the snail *Biomphalaria glabrata*. *Fish Shellfish Immunol* (2019) **88**:301–307. doi:10.1016/j.fsi.2019.02.065

6. Allan ERO, Blouin MS. Heat shock increases hydrogen peroxide release from circulating hemocytes of the snail *Biomphalaria glabrata*. *Fish Shellfish Immunol* (2020) **105**:203–208. doi:10.1016/j.fsi.2020.07.029

7. Baron OL, van West P, Industri B, Ponchet M, Dubreuil G, Gourbal B, Reichhart JM, Coustau C. Parental transfer of the antimicrobial protein LBP/BPI protects *Biomphalaria glabrata* eggs against oomycete Infections. *PLoS Pathog* (2013) **9**:1–10. doi:10.1371/journal.ppat.1003792

8. Baron OL, Deleury E, Reichhart J-M, Coustau C. The LBP/BPI multigenic family in invertebrates: evolutionary history and evidences of specialization in mollusks. *Dev Comp Immunol* (2016) **57**:20–30. doi:10.1016/j.dci.2015.11.006

9. Bender RC, Goodall CP, Blouin MS, Bayne CJ. Variation in expression of *Biomphalaria glabrata SOD1*: a potential controlling factor in susceptibility/resistance to *Schistosoma mansoni*. *Dev Comp Immunol* (2007) **31**:874–878. doi:10.1016/j.dci.2006.12.005

10. Bouchut A, Roger E, Coustau C, Gourbal B, Mitta G. Compatibility in the *Biomphalaria glabrata/Echinostoma caproni* model: potential involvement of adhesion genes. *Int J Parasitol* (2006) **36**:175–184. doi:10.1016/j.ijpara.2005.09.009

11. Bouchut A, Sautiere PE, Coustau C, Mitta G. Compatibility in the *Biomphalaria glabrata/Echinostoma caproni* model: potential involvement of proteins from hemocytes revealed by a proteomic approach. *Acta Trop* (2006) **98**:234–246. doi:10.1016/j.actatropica.2006.05.007

12. Bouchut A, Coustau C, Gourbal B, Mitta G. Compatibility in the *Biomphalaria glabrata/Echinostoma caproni* model: new candidate genes evidenced by a suppressive subtractive hybridization approach. *Parasitology* (2007) **134**:575–88. doi:10.1017/S0031182006001673

13. Bridger JM, Brindley PJ, Knight M. The snail *Biomphalaria glabrata* as a model to interrogate the molecular basis of complex human diseases. *PLoS Negl Trop Dis* (2018) **12**:e0006552. doi:10.1371/journal.pntd.0006552

14. da Silva Cantinha R, Borrely SI, Oguiura N, de Bragança Pereira CA, Rigolon MM, Nakano E. HSP70 expression in *Biomphalaria glabrata* snails exposed to cadmium. *Ecotoxicol Environ Saf* (2017) **140**:18–23. doi:10.1016/j.ecoenv.2017.02.026

15. de Melo ES, Brayner FA, Junior NCP, França IRS, Alves LC. Investigation of defense response and immune priming in *Biomphalaria glabrata* and *Biomphalaria straminea*, two species with different susceptibility to *Schistosoma mansoni*. *Parasitol Res* (2020) **119**:189–201. doi:10.1007/s00436-019-06495-4

16. Deleury E, Dubreuil G, Elangovan N, Wajnberg E, Reichhart JM, Gourbal B, Duval D, Baron OL, Gouzy J, Coustau C. Specific versus non-specific immune responses in an invertebrate species evidenced by a comparative *de novo* sequencing study. *PLoS One* (2012) **7**: doi:10.1371/journal.pone.0032512

17. Dheilly NM, Duval D, Mouahid G, Emans R, Allienne JF, Galinier R, Genthon C, Dubois E, Du Pasquier L, Adema CM, et al. A family of variable immunoglobulin and lectin domain containing molecules in the snail *Biomphalaria glabrata*. *Dev Comp Immunol* (2015) **48**:234–243. doi:10.1016/j.dci.2014.10.009

18. Dinguirard N, Cavalcanti MGS, Wu X-J, Bickham-Wright U, Sabat G, Yoshino TP. Proteomic analysis of *Biomphalaria glabrata* hemocytes during in vitro encapsulation of *Schistosoma mansoni* sporocysts. *Front Immunol* (2018) **9**:1–17. doi:10.3389/fimmu.2018.02773

19. Fogarty CE, Zhao M, McManus DP, Duke MG, Cummins SF, Wang T. Comparative study of excretory–secretory proteins released by *Schistosoma mansoni*-resistant, susceptible and naïve *Biomphalaria glabrata*. *Parasit Vectors* (2019) **12**:1–17. doi:10.1186/s13071-019-3708-0

20. Galinier R, Portela J, Moné Y, Allienne JF, Henri H, Delbecq S, Mitta G, Gourbal B, Duval D. Biomphalysin, a new *β* pore-forming toxin involved in *Biomphalaria glabrata* immune defense against *Schistosoma mansoni*. *PLoS Pathog* (2013) **9**: doi:10.1371/journal.ppat.1003216

21. Galinier R, Roger E, Moné Y, Duval D, Portet A, Pinaud S, Chaparro C, Grunau C, Genthon C, Dubois E, et al. A multistrain approach to studying the mechanisms underlying compatibility in the interaction between *Biomphalaria glabrata* and *Schistosoma mansoni*. *PLoS Negl Trop Dis* (2017) **11**:e0005398. doi:10.1371/journal.pntd.0005398

22. Goodall CP, Bender RC, Broderick EJ, Bayne CJ. Constitutive differences in *Cu/Zn* superoxide dismutase mRNA levels and activity in hemocytes of *Biomphalaria glabrata* (Mollusca) that are either susceptible or resistant to *Schistosoma mansoni* (Trematoda). *Mol Biochem Parasitol* (2004) **137**:321–328. doi:10.1016/j.molbiopara.2004.06.011

23. Granath WO, Connors VA, Tarleton RL. Interleukin 1 activity in haemolymph from strains of the snail *Biomphalaria glabrata* varying in susceptibility to the human blood fluke, *Schistosoma mansoni*: presence, differential expression, and biological function. *Cytokine* (1994) **6**:21–27. doi:10.1016/1043-4666(94)90003-5

24. Guillou F, Mitta G, Dissous C, Pierce R, Coustau C. Use of individual polymorphism to validate potential functional markers: case of a candidate lectin (BgSel) differentially expressed in susceptible and resistant strains of *Biomphalaria glabrata*. *Comp Biochem Physiol - B Biochem Mol Biol* (2004) **138**:175–181. doi:10.1016/j.cbpc.2004.03.010

25. Guillou F, Mitta G, Galinier R, Coustau C. Identification and expression of gene transcripts generated during an anti-parasitic response in *Biomphalaria glabrata*. *Dev Comp Immunol* (2007) **31**:657–671. doi:10.1016/j.dci.2006.10.001

26. Hanelt B, Lun CM, Adema CM. Comparative ORESTES-sampling of transcriptomes of immune-challenged *Biomphalaria glabrata* snails. *J Invertebr Pathol* (2008) **99**:192–203. doi:10.1016/j.jip.2008.06.002

27. Hanington PC, Lun C-M, Adema CM, Loker ES. Time series analysis of the transcriptional responses of *Biomphalaria glabrata* throughout the course of intramolluscan development of *Schistosoma mansoni* and *Echinostoma paraensei*. *Int J Parasitol* (2010) **40**:819–831. doi:10.1016/j.ijpara.2009.12.005

28. Hanington PC, Forys MA, Loker ES. A somatically diversified defense factor, FREP3, is a determinant of snail resistance to schistosome infection. *PLoS Negl Trop Dis* (2012) **6**: doi:10.1371/journal.pntd.0001591

29. Hathaway JJM, Adema CM, Stout BA, Mobarak CD, Loker ES. Identification of protein components of egg masses indicates parental investment in immunoprotection of offspring by *Biomphalaria glabrata* (Gastropoda, Mollusca). *Dev Comp Immunol* (2010) **34**:425–435. doi:10.1016/j.dci.2009.12.001.Identification

30. Hertel LA, Adema CM, Loker ES. Differential expression of *FREP* genes in two strains of *Biomphalaria glabrata* following exposure to the digenetic trematodes *Schistosoma mansoni* and *Echinostoma paraensei*. *Dev Comp Immunol* (2005) **29**:295–303. doi:10.1016/j.dci.2004.08.003

31. Ittiprasert W, Nene R, Miller A, Raghavan N, Lewis F, Hodgson J, Knight M. *Schistosoma manson*i infection of juvenile *Biomphalaria glabrata* induces a differential stress response between resistant and susceptible snails. *Exp Parasitol* (2009) **123**:203–211. doi:10.1016/j.exppara.2009.07.015

32. Ittiprasert W, Miller A, Myers J, Nene V, El-Sayed NM, Knight M. Identification of immediate response genes dominantly expressed in juvenile resistant and susceptible *Biomphalaria glabrata* snails upon exposure to *Schistosoma mansoni*. *Mol Biochem Parasitol* (2010) **169**:27–39. doi:10.1016/j.molbiopara.2009.09.009

33. Jiang Y, Loker ES, Zhang S-M. In vivo and in vitro knockdown of *FREP2* gene expression in the snail *Biomphalaria glabrata* using RNA interference. *Dev Comp Immunol* (2006) **30**:855–866. doi:10.1016/j.dci.2005.12.004

34. Jones CS, Lockyer AE, Rollinson D, Noble LR. Molecular approaches in the study of *Biomphalaria glabrata* – *Schistosoma mansoni* interactions: linkage analysis and gene expression profiling. *Parasitology* (2001) **123**:181–196. doi:10.1017/S0031182001008174

35. Jung Y, Nowak TS, Zhang S-M, Hertel LA, Loker ES, Adema CM. Manganese superoxide dismutase from *Biomphalaria glabrata*. *J Invertebr Pathol* (2005) **90**:59–63. doi:10.1016/j.jip.2005.06.014

36. Kaur S, Baynes A, Lockyer AE, Routledge EJ, Jones CS, Noble LR, Jobling S. Steroid androgen exposure during development has no effect on reproductive physiology of *Biomphalaria glabrata*. *PLoS One* (2016) **11**:1–22. doi:10.1371/journal.pone.0159852

37. Knight M, Raghavan N, Goodall C, Cousin C, Ittiprasert W, Sayed A, Miller A, Williams DL, Bayne CJ. *Biomphalaria glabrata* peroxiredoxin: effect of *Schistosoma mansoni* infection on differential gene regulation. *Mol Biochem Parasitol* (2009) **167**:20–31. doi:10.1016/j.molbiopara.2009.04.002

38. Knight M, Miller A, Liu Y, Scaria P, Woodle M, Ittiprasert W. Polyethyleneimine (PEI) mediated sirna gene silencing in the *Schistosoma mansoni* snail host, *Biomphalaria glabrata*. *PLoS Negl Trop Dis* (2011) **5**: doi:10.1371/journal.pntd.0001212

39. Knight M, Elhelu O, Smith M, Haugen B, Miller A, Raghavan N, Wellman C, Cousin C, Dixon F, Mann V, et al. Susceptibility of snails to infection with schistosomes is influenced by temperature and expression of heat shock proteins. *Epidemiol Open Access* (2015) **05**: doi:10.4172/2161-1165.1000189

40. Knight M, Ittiprasert W, Arican-Goktas HD, Bridger JM. Epigenetic modulation, stress and plasticity in susceptibility of the snail host, *Biomphalaria glabrata*, to *Schistosoma mansoni* infection. *Int J Parasitol* (2016) **46**:389–394. doi:10.1016/j.ijpara.2016.03.003

41. Larson MK, Bender RC, Bayne CJ. Resistance of *Biomphalaria glabrata* 13-16-R1 snails to *Schistosoma mansoni* PR1 is a function of haemocyte abundance and constitutive levels of specific transcripts in haemocytes. *Int J Parasitol* (2014) **44**:343–353. doi:10.1016/j.ijpara.2013.11.004

42. Lassalle D, Tetreau G, Pinaud S, Galinier R, Crickmore N, Gourbal B, Duval D. Glabralysins, potential new β-pore-forming toxin family members from the schistosomiasis vector snail *Biomphalaria glabrata*. *Genes (Basel)* (2020) **11**:65. doi:10.3390/genes11010065

43. Lehr T, Frank S, Natsuka S, Geyer H, Beuerlein K, Doenhoff MJ, Hase S, Geyer R. N-Glycosylation patterns of hemolymph glycoproteins from *Biomphalaria glabrata* strains expressing different susceptibility to *Schistosoma mansoni* infection. *Exp Parasitol* (2010) **126**:592–602. doi:10.1016/j.exppara.2010.06.031

44. Li H, Hambrook JR, Pila EA, Gharamah AA, Fang J, Wu X, Hanington P. Coordination of humoral immune factors dictates compatibility between *Schistosoma mansoni* and *Biomphalaria glabrata*. *Elife* (2020) **9**: doi:10.7554/eLife.51708

45. Li H, Gharamah AA, Hambrook JR, Wu X, Hanington PC. Single-cell RNA-seq profiling of individual *Biomphalaria glabrata* immune cells with a focus on immunologically relevant transcripts. *Immunogenetics* (2021) doi:10.1007/s00251-021-01236-3

46. Lockyer AE, Jones CS, Noble LR, Rollinson D. Use of differential display to detect changes in gene expression in the intermediate snail host *Biomphalaria glabrata* upon infection with *Schistosoma mansoni*. *Parasitology* (2000) **120**:399–407. doi:10.1017/S0031182099005624

47. Lockyer AE, Noble LR, Rollinson D, Jones CS. *Schistosoma mansoni*: resistant specific infection-induced gene expression in *Biomphalaria glabrata* identified by fluorescent-based differential display. *Exp Parasitol* (2004) **107**:97–104. doi:10.1016/j.exppara.2004.04.004

48. Lockyer AE, Spinks J, Noble LR, Rollinson D, Jones CS. Identification of genes involved in interactions between *Biomphalaria glabrata* and *Schistosoma mansoni* by suppression subtractive hybridization. *Mol Biochem Parasitol* (2007) **151**:18–27. doi:10.1016/j.molbiopara.2006.09.009

49. Lockyer AE, Spinks JN, Walker AJ, Kane RA, Noble LR, Rollinson D, Dias-Neto E, Jones CS. *Biomphalaria glabrata* transcriptome: identification of cell-signalling, transcriptional control and immune-related genes from open reading frame expressed sequence tags (ORESTES). *Dev Comp Immunol* (2007) **31**:763–782. doi:10.1016/j.dci.2006.11.004

50. Lockyer AE, Spinks J, Kane RA, Hoffmann KF, Fitzpatrick JM, Rollinson D, Noble LR, Jones CS. Biomphalaria glabrata transcriptome: cDNA microarray profiling identifies resistant- and susceptible-specific gene expression in haemocytes from snail strains exposed to *Schistosoma mansoni*. *BMC Genomics* (2008) **9**: doi:10.1186/1471-2164-9-634

51. Lockyer AE, Emery AM, Kane RA, Walker AJ, Mayer CD, Mitta G, Coustau C, Adema CM, Hanelt B, Rollinson D, et al. Early differential gene expression in haemocytes from resistant and susceptible *Biomphalaria glabrata* strains in response to *Schistosoma mansoni*. *PLoS One* (2012) **7**:e51102. doi:10.1371/journal.pone.0051102

52. Lu L, Loker ES, Zhang S-M, Buddenborg SK, Bu L. Genome-wide discovery, and computational and transcriptional characterization of an AIG gene family in the freshwater snail *Biomphalaria glabrata*, a vector for *Schistosoma mansoni*. *BMC Genomics* (2020) **21**:190. doi:10.1186/s12864-020-6534-z

53. Lu L, Loker ES, Adema CM, Zhang S-M, Bu L. Genomic and transcriptional analysis of genes containing fibrinogen and IgSF domains in the schistosome vector *Biomphalaria glabrata*, with emphasis on the differential responses of snails susceptible or resistant to *Schistosoma mansoni*. *PLoS Negl Trop Dis* (2020) **14**:e0008780. doi:10.1371/journal.pntd.0008780

54. Mendes TMF, Carrilho E, Afonso AJPFS, Galinaro CA, Cabral FJ, Allegretti SM. Proteomic, metabolic and immunological changes in *Biomphalaria glabrata* infected with *Schistosoma mansoni.* *Int J Parasitol* (2019) **49**:1049–1060. doi:https://doi.org/10.1016/j.ijpara.2019.08.001

55. Mendes TMF, Carrilho E, Galinaro CA, Cabral FJ, Allegretti SM. *Biomphalaria glabrata* infected with *Angiostrongylus cantonensis*: proteomic changes in the snail host. *Acta Trop* (2020) **212**: doi:10.1016/j.actatropica.2020.105684

56. Mitta G, Galinier R, Tisseyre P, Allienne J-F, Girerd-Chambaz Y, Guillou F, Bouchut A, Coustau C. Gene discovery and expression analysis of immune-relevant genes from *Biomphalaria glabrata* hemocytes. *Dev Comp Immunol* (2005) **29**:393–407. doi:10.1016/j.dci.2004.10.002

57. Moné Y, Ribou AC, Cosseau C, Duval D, Théron A, Mitta G, Gourbal B. An example of molecular co-evolution: reactive oxygen species (ROS) and ROS scavenger levels in *Schistosoma mansoni/Biomphalaria glabrata* interactions. *Int J Parasitol* (2011) **41**:721–730. doi:10.1016/j.ijpara.2011.01.007

58. Myers J, Ittiprasert W, Raghavan N, Miller A, Knight M. Differences in cysteine protease activity in *Schistosoma mansoni*-resistant and -susceptible *Biomphalaria glabrata* and characterization of the hepatopancreas Cathepsin B full-length cDNA. *J Parasitol* (2008) **94**:659–668. doi:10.1645/ge-1410r.1

59. Nelson MK, Cruz BC, Buena KL, Nguyen H, Sullivan JT. Effects of abnormal temperature and starvation on the internal defense system of the schistosome-transmitting snail *Biomphalaria glabrata*. *J Invertebr Pathol* (2016) **138**:18–23. doi:10.1016/j.jip.2016.05.009

60. Nowak TS, Woodards AC, Jung Y, Adema CM, Loker ES. Identification of transcripts generated during the response of resistant *Biomphalaria glabrata* to *Schistosoma mansoni* infection using suppression subtractive hybridization. *J Parasitol* (2004) **90**:1034–40. doi:10.1645/GE-193R1

61. Pila EA, Gordy MA, Phillips VK, Kabore AL, Rudko SP, Hanington PC. Endogenous growth factor stimulation of hemocyte proliferation induces resistance to *Schistosoma mansoni* challenge in the snail host. *Proc Natl Acad Sci* (2016) **113**:5305–5310. doi:10.1073/pnas.1521239113

62. Pila EA, Tarrabain M, Kabore AL, Hanington PC. A novel Toll-Like receptor (TLR) influences compatibility between the gastropod *Biomphalaria glabrata*, and the digenean trematode *Schistosoma mansoni*. *PLoS Pathog* (2016) **12**:e1005513. doi:10.1371/journal.ppat.1005513

63. Pinaud S, Portela J, Duval D, Nowacki FC, Olive M-A, Allienne J-F, Galinier R, Dheilly NM, Kieffer-Jaquinod S, Mitta G, et al. A shift from cellular to humoral responses contributes to innate immune memory in the vector snail *Biomphalaria glabrata*. *PLoS Pathog* (2016) **12**:e1005361. doi:10.1371/journal.ppat.1005361

64. Pinaud S, Portet A, Allienne JF, Belmudes L, Saint-Beat C, Arancibia N, Galinier R, Du Pasquier L, Duval D, Gourbal B. Molecular characterisation of immunological memory following homologous or heterologous challenges in the schistosomiasis vector snail, *Biomphalaria glabrata*. *Dev Comp Immunol* (2019) **92**:238–252. doi:10.1016/j.dci.2018.12.001

65. Pinaud S, Tetreau G, Poteaux P, Galinier R, Chaparro C, Lassalle D, Portet A, Simphor E, Gourbal B, Duval D. New insights into biomphalysin gene family diversification in the vector snail *Biomphalaria glabrata*. *Front Immunol* (2021) **12**:1–19. doi:10.3389/fimmu.2021.635131

66. Portet A, Galinier R, Pinaud S, Portela J, Nowacki F, Gourbal B, Duval D. BgTEP: an antiprotease involved in innate immune sensing in *Biomphalaria glabrata*. *Front Immunol* (2018) **9**: doi:10.3389/fimmu.2018.01206

67. Portet A, Pinaud S, Chaparro C, Galinier R, Dheilly NM, Portela J, Charriere GM, Allienne J-F, Duval D, Gourbal B. Sympatric versus allopatric evolutionary contexts shape differential immune response in *Biomphalaria / Schistosoma* interaction. *PLOS Pathog* (2019) **15**:e1007647. doi:10.1371/journal.ppat.1007647

68. Portet A, Toulza E, Lokmer A, Huot C, Duval D, Galinier R, Gourbal B. Experimental infection of the *Biomphalaria glabrata* vector snail by *Schistosoma mansoni* parasites drives snail microbiota dysbiosis. *Microorganisms* (2021) **9**:1084. doi:10.3390/microorganisms9051084

69. Queiroz FR, Silva LM, Jeremias W de J, Babá ÉH, Caldeira RL, Coelho PMZ, Gomes M de S. Differential expression of small RNA pathway genes associated with the *Biomphalaria glabrata/Schistosoma mansoni* interaction. *PLoS One* (2017) **12**:e0181483. doi:10.1371/journal.pone.0181483

70. Raghavan N, Miller AN, Gardner M, FitzGerald PC, Kerlavage AR, Johnston DA, Lewis FA, Knight M. Comparative gene analysis of *Biomphalaria glabrata* hemocytes pre- and post-exposure to miracidia of *Schistosoma mansoni*. *Mol Biochem Parasitol* (2003) **126**:181–191. doi:10.1016/S0166-6851(02)00272-4

71. Smith M, Yadav S, Fagunloye OG, Pels NA, Horton DA, Alsultan N, Borns A, Cousin C, Dixon F, Mann VH, et al. PIWI silencing mechanism involving the retrotransposon nimbus orchestrates resistance to infection with *Schistosoma mansoni* in the snail vector, *Biomphalaria glabrata*. *PLoS Negl Trop Dis* (2021) **15**:e0009094. doi:10.1371/journal.pntd.0009094

72. Tennessen JA, Bollmann SR, Peremyslova E, Kronmiller BA, Sergi C, Hamali B, Blouin MS. Clusters of polymorphic transmembrane genes control resistance to schistosomes in snail vectors. *Elife* (2020) **9**:1–16. doi:10.7554/eLife.59395

73. Tunholi-Alves VM, Tunholi VM, Gôlo P, Lustrino D, Maldonado A, Bittencourt VREP, Rodrigues M de L de A, Pinheiro J. Lipid levels in *Biomphalaria glabrata* infected with different doses of *Echinostoma paraensei* miracidia. *Exp Parasitol* (2011) **128**:212–216. doi:10.1016/j.exppara.2011.03.009

74. Vergote D, Bouchut A, Sautière PE, Roger E, Galinier R, Rognon A, Coustau C, Salzet M, Mitta G. Characterisation of proteins differentially present in the plasma of *Biomphalaria glabrata* susceptible or resistant to *Echinostoma caproni*. *Int J Parasitol* (2005) **35**:215–224. doi:10.1016/j.ijpara.2004.11.006

75. Zahoor Z, Davies AJ, Kirk RS, Rollinson D, Walker AJ. Larval excretory-secretory products from the parasite *Schistosoma mansoni* modulate HSP70 protein expression in defence cells of its snail host, *Biomphalaria glabrata*. *Cell Stress Chaperones* (2010) **15**:639–650. doi:10.1007/s12192-010-0176-z

76. Zahoor Z, Lockyer AE, Davies AJ, Kirk RS, Emery AM, Rollinson D, Jones CS, Noble LR, Walker AJ. Differences in the gene expression profiles of haemocytes from schistosome-susceptible and -resistant *Biomphalaria glabrata* exposed to *Schistosoma mansoni* excretory-secretory products. *PLoS One* (2014) **9**:e93215. doi:10.1371/journal.pone.0093215

77. Zhang S-M, Loker ES. The *FREP* gene family in the snail *Biomphalaria glabrata*: additional members, and evidence consistent with alternative splicing and *FREP* retrosequences. *Dev Comp Immunol* (2003) **27**:175–187. doi:10.1016/S0145-305X(02)00091-5

78. Zhang S-M, Zeng Y, Loker ES. Characterization of immune genes from the schistosome host snail *Biomphalaria glabrata* that encode peptidoglycan recognition proteins and gram-negative bacteria binding protein. *Immunogenetics* (2007) **59**:883–898. doi:10.1007/s00251-007-0245-3

79. Zhang S-M, Nian H, Zeng Y, DeJong RJ. Fibrinogen-bearing protein genes in the snail *Biomphalaria glabrata*: characterization of two novel genes and expression studies during ontogenesis and trematode infection. *Dev Comp Immunol* (2008) **32**:1119–1130. doi:10.1016/j.dci.2008.03.001

80. Zhang S-M, Zeng Y, Loker ES. Expression profiling and binding properties of fibrinogen-related proteins (FREPs), plasma proteins from the schistosome snail host *Biomphalaria glabrata*. *Innate Immun* (2008) **14**:175–89. doi:10.1177/1753425908093800

81. Zhang S-M, Nian H, Wang B, Loker ES, Adema CM. Schistosomin from the snail *Biomphalaria glabrata*: expression studies suggest no involvement in trematode-mediated castration. *Mol Biochem Parasitol* (2009) **165**:79–86. doi:10.1016/j.molbiopara.2009.01.006

82. Zhang S-M, Coultas KA. Identification and characterization of five transcription factors that are associated with evolutionarily conserved immune signaling pathways in the schistosome-transmitting snail *Biomphalaria glabrata*. *Mol Immunol* (2011) **48**:1868–81. doi:10.1016/j.molimm.2011.05.017

83. Zhang S-M, Buddenborg SK, Adema CM, Sullivan JT, Loker ES. Altered gene expression in the schistosome-transmitting snail *Biomphalaria glabrata* following exposure to niclosamide, the active ingredient in the widely used molluscicide bayluscide. *PLoS Negl Trop Dis* (2015) **9**:e0004131. doi:10.1371/journal.pntd.0004131

84. Zhang S-M, Loker ES, Sullivan JT. Pathogen-associated molecular patterns activate expression of genes involved in cell proliferation, immunity and detoxification in the amebocyte-producing organ of the snail *Biomphalaria glabrata*. *Dev Comp Immunol* (2016) **56**:25–36. doi:10.1016/j.dci.2015.11.008
